# Efficacy of non-invasive brain stimulation on cognitive and motor functions in multiple sclerosis: A systematic review and meta-analysis

**DOI:** 10.3389/fneur.2023.1091252

**Published:** 2023-01-26

**Authors:** Shuiyan Li, Qi Zhang, Shuqi Zheng, Gege Li, Shilin Li, Longlong He, Yuting Zeng, Ling Chen, Shuping Chen, Xiaoyan Zheng, Jihua Zou, Qing Zeng

**Affiliations:** ^1^School of Rehabilitation Sciences, Southern Medical University, Guangzhou, China; ^2^Department of Rehabilitation Medicine, Zhujiang Hospital, Southern Medical University, Guangzhou, China; ^3^Faculty of Health and Social Sciences, The Hong Kong Polytechnic University, Hong Kong, China

**Keywords:** non-invasive brain stimulation, multiple sclerosis, cognition function, motor function, meta-analysis

## Abstract

**Objective:**

In this study, we aimed to investigate the effects of non-invasive brain stimulation (NIBS) on cognitive and motor functions in patients with multiple sclerosis (pwMS).

**Methods:**

A literature search was performed in the Cochrane Library, Embase, PubMed, Web of Science, Medline, CNKI, and Wan fang. The time interval used for database construction was up to December 2022, and the language was not limited. The collected trials were subsequently screened, the data were extracted, the quality was evaluated, and the effect sizes were computed using STATA/MP Version 13 for outcome analysis. Standard mean difference (SMD) and 95% confidence interval (CI) were calculated for domain of interest.

**Results:**

In total, 17 articles that examined 364 patients with multiple sclerosis were included in this analysis. Non-invasive brain stimulation did not improve the overall cognitive function [SMD = 0.18, 95% CI (−0.32, 0.69), *P* = 0.475] but helped improve motor function in patients [SMD = 0.52, 95% CI (0.19, 0.85), *P* = 0.002]. Moreover, this study specifically indicated that non-invasive brain stimulation improved alerting [SMD = 0.68, 95% CI (0.09, 1.26), *P* = 0.02], whereas non-invasive brain stimulation intervention improved motor function in patients aged <45 years [SMD = 0.67, 95% CI (0.23, 1.10), *P* = 0.003] and in patients with expanded disability status scale scores (EDSS) <3.5 [SMD = 0.82, 95% CI (0.22, 1.42), *P* = 0.007]. In particular, NIBS contributed to the improvement of spasticity in pwMS [SMD = 0.68, 95% CI (0.13, 1.23), *P* = 0.015].

**Conclusion:**

These results of this present study provide evidence that non-invasive brain stimulation could improve alertness in pwMS. Furthermore, NIBS may help pwMS with motor function and those who are under 45 years of age or with EDSS < 3.5 improve their motor function. For the therapeutic use of NIBS, we recommend applying transcranial magnetic stimulation as an intervention and located on the motor cortex M1 according to the subgroup analysis of motor function. These findings warrant verification.

**Systematic review registration:**

https://www.crd.york.ac.uk/PROSPERO/, identifier CRD42022301012.

## 1. Introduction

Multiple sclerosis (MS) is a chronic, paroxysmal, autoimmune disease that is characterized by demyelination and axonal degeneration of the central nervous system (1). Clinically, multiple sclerosis is classified into primary progressive type, secondary progressive type, and relapsing remitting type (2). The prevalence of MS, which is correlated with region, often exhibits a trend of rapid growth worldwide, with North America and Europe having the highest prevalence (3, 4). Usually, the clinical manifestations of MS include various cognitive impairments (such as attention deficit and executive dysfunction), motor impairments (such as spasms and tremors), sensory abnormalities (such as pain), visual impairments (such as diplopia and optic nerve dysfunction), and behavioral abnormalities (5). Moreover, among the symptoms described above, cognitive impairments affect 45–70% of patients with MS (pwMS) (6), and cognitive impairment may appear early in the course of the disease and worsen as the disease progresses (7), resulting in diminished quality of life and social dysfunction (8). Besides, motor dysfunction, which can be caused by a variety of issues (such as spasms, muscle weakness, abnormal walking mechanics, balance, or exhaustion) (9), can affect roughly 80% of pwMS and has major consequences for their personal and professional lives (10). In addition, cognitive dysfunction in pwMS, which increases the risk of falls and impairs motor function, has been closely associated with motor dysfunction (11). Consequently, it is of particular importance to treat the cognitive and motor functions in pwMS, in order to improve their quality of life and well-being.

In MS, drugs can reduce the number of flare-ups and slow the natural course of the disease. However, some patients experience side effects and thus seek complementary and alternative treatments to strike an appropriate balance between drug efficacy and safety (12). Furthermore, considering the complexity of the clinical symptoms of this condition, additional personalized and multi-selective treatments for pwMS need to be identified. Recently, non-invasive brain stimulation (NIBS) has attracted significant attention in the academic world (9) as a complementary method for treating neurological diseases. In general, NIBS is known to induce excitatory changes *via* the application of electrical and/or magnetic energy in the underlying cerebral cortex in a non-invasive manner and may induce long-lasting neuroplasticity changes (13). Clinically, transcranial direct current stimulation (tDCS) and repetitive transcranial magnetic stimulation (rTMS) are the most widely used NIBS techniques (14, 15). To date, researchers have experimentally explored the effects of the NIBS technology on the cognitive function and motor function of pwMS. However, because of the different experimental designs, the experimental results remain controversial, to some extent. This calls for a more effective study of the effects of NIBS in MS. To the best of our knowledge, previous evidence-based medical studies have explored the effects of NIBS applied to MS, particularly on pain (16) and fatigue (17), whereas no such evidence has been reported regarding cognitive and motor functions. Notably, there are no guidelines for the therapeutic use of NIBS in MS (18), and the effect of its clinical application remains to be researched and discussed.

Given the uncertainty of the effect of its clinical use, here we searched and screened clinical randomized trials on the effects of existing NIBS techniques on pwMS, aiming to explore the impact of NIBS on the cognitive and motor functions of pwMS and to draw evidence-based medical conclusions to provide guidance for its clinical practice and applications.

## 2. Materials and methods

The study protocol was designed in accordance with the Preferred Reporting for Systematic Reviews and Meta-analysis (PRISMA) statement (19) and was prospectively registered in PROSPERO (CRD42022301012).

### 2.1. Search strategy

According to the PRISMA guidelines and Population, Intervention, Comparison, Outcome, and Study (PICOS) design, two authors conducted a systematic search of the existing literature on this topic in the Cochrane Library (from 1996), Embase (from 1980), PubMed (from 1950), Web of Science (from 1986), Medline (from 1950), China National Knowledge Infrastructure (CNKI) (from 1994), and Wan fang database (from 1998), from the inception of each of these databases to 20 December 2022. The selected search terms included transcranial direct current stimulation, transcranial random noise stimulation, transcranial electrical stimulation, transcranial magnetic stimulation, theta burst stimulation, non-invasive brain stimulation, multiple sclerosis, disseminated sclerosis, MS, cognitive functions, and motor function. The literature language was unlimited. The specific search strategy used for each database is available in the Supplementary material 1.

### 2.2. Literature screening

The inclusion and exclusion criteria of the literature were jointly formulated by the three authors, and the screening process was mainly completed by two of the authors independently. In case of differences of opinion between the two authors, the third author would also intervene to complete the discussion.

### 2.3. Inclusion and exclusion criteria

Studies that met the following criteria were considered for inclusion in our analysis: (1) subjects: adult men and women (18 years of age or older) with clinically confirmed MS and all types of MS, such as primary progressive type, secondary progressive type, and relapsing remitting type, stable physical and pharmacological therapies since at least 1 month; (2) intervention measures: the intervention methods were transcranial magnetic stimulation, transcranial direct current stimulation, transcranial alternating current stimulation, and transcranial random noise stimulation; (3) outcome measures: the study results (primary or secondary) included cognitive- or motor-related measures (Attention Network Test, number symbol matching test, individual variation index, timed 25-foot walk test, 5-repeat sit-up test, multiple sclerosis walking scale, and 10-minute walk test); and (4) study type: clinical randomized controlled trial. The exclusion criteria were as follows: (1) trials including individuals with other neurological or non-neurological comorbidities that may affect motor and cognitive function; (2) the number of participants in the trial was <5; (3) uncompleted and unpublished experiments or the paper was part of the study protocol; and (4) the data of the trial were not clear or missing and did not meet the data requirements of the test.

### 2.4. Data extraction and quality assessment

Two of the authors independently screened the title and abstract and selected the papers in strict compliance with the inclusion criteria; subsequently, the selected papers were read in full, and the reasons for exclusion were recorded and documented. Inconsistent opinions were resolved *via* a discussion, with the third author joining in. For the included studies, two of the authors extracted the information according to the PICOS guidelines, which have been commonly used in evidence-based medicine to construct clinical questions. This included patient information (number, age, sex, type of MS, and time from symptom onset to diagnosis); intervention information (site, parameters, and dose); comparative treatment measures (conventional cognitive training and motor training); outcome (Attention Network Test, 10-min walking test, and other test results after clinical intervention using non-invasive brain stimulation); and study design (randomized controlled trial).

The methodological quality of all eligible studies was determined by two of the authors based on the risk assessed using a Cochrane's risk of bias tool, which was carried out on seven aspects, including grouping method, allocation scheme, implementation blindness, outcome blindness, outcome integrity, selective analysis, and other possible biases.

### 2.5. Statistical analysis

The STATA/MP Version 13 software and fixed-/random-effects models were used for the statistical analysis of the pooled data. To compare the treatments, the effect size estimate and 95% confidence interval were used. Heterogeneity was assessed using Higgins' *I*^2^ statistics, with *I*^2^ ≥ 50% indicating substantial heterogeneity. Subgroup analyses (e.g., different outcome indicators and age groups) were performed to reduce heterogeneity by grouping the studies that met the inclusion conditions; alternatively, a sensitivity analysis was performed to explore the source of the heterogeneity by excluding each literature item. Notably, if the outcome index in the included literature indicates that the parameters of the same impact vary in opposite directions, we processed them so that a parameter >0 indicates a positive effect.

A funnel plot was also used to evaluate publication bias; according to Cochrane guidelines (20), monitoring of publication bias is recommended when ten or more papers are included in the analysis. The funnel plot was used to measure the publication bias of individual studies based on the pooled estimate. If the funnel plot was symmetrical, the probability of bias was low; in contrast, if it was asymmetrical, the probability of bias was high.

## 3. Results

### 3.1. Literature evaluation

**Figure 1:**
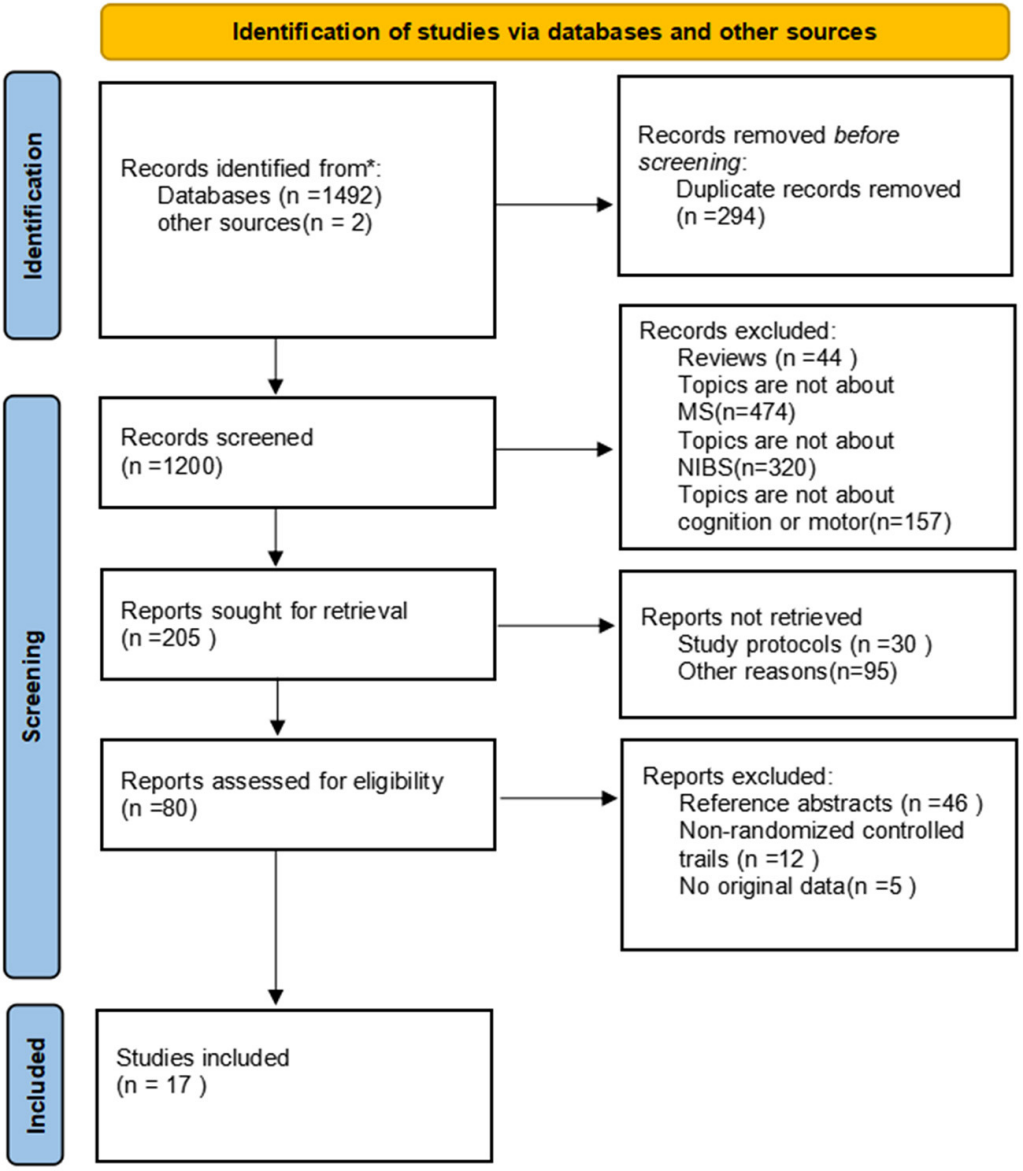
Flow diagram of literature screening.

In total, 1494 articles were retrieved from the literature, and 294 duplicated articles were removed. There were 44 reviews and 951 articles irrelevant to the research topic, which were excluded by reading the title and abstract. As 30 articles were study protocols and 95 records were removed for other reasons, the full texts of the remaining 80 studies were read. Among those studies, 46 articles that were reference abstracts and 12 articles reporting non-clinical randomized trials were excluded, another 5 reports were excluded because there are no original data. Finally, 17 English articles were yielded (21–37). Of these, 10 reported cognition outcomes (21–30), eight investigated motor function (30–37), as an article (30) reported both cognition and motor outcomes. The specific literature screening process used here is shown in Figure 1. Tables 1, 2, respectively, list the characteristics of each included article based on different topics and the characteristics of participants are presented in Table 3. It can be observed visually that a total of 364 pwMS were included in this study, of which mostly were female patients, accounting for more than 70% of the cohort, which conforms to the epidemiological characteristics of MS (38). Few studies were performed in China, which is related to the epidemiological characteristics of MS in China (39, 40). In recent years, it has been discovered that the incidence of MS has been increasing overall, including in low-incidence areas (4), and the research on MS in China cannot be ignored. Among all subtypes included patients with relapsing–remitting MS type were noted to be the most frequent with at least 213 RR among 364 pwMS. Different experimental designs were used in the included studies, including five cross-over designs (24–28), 12 randomized parallel trials (21–23, 29–37), two open-label experiments (21, 35), and a study (21) that had no direct mention of the use of parallel controlled trials (we classified it into the parallel controlled trials category according to the experimental design after reading the full text).

**Table 1:** Included cognitive-related studies characteristics.

**Study**	**Year**	**Study design**	**Country**	**Sample Size**		**Interventions**		**Sex (Female: Male)**		**Intervention site**	**Stimulation intensity**	**Stimulation time**	**Outcome indicator**
				**Exp**	**Ctrl**	**Exp**	**Ctrl**	**Exp**	**Ctrl**				
Charvet et al. (21)	2018	Parallel	USA	25	20	RS-tDCS+CT	CT	21:4	13:7	Anode: Left dorsolateral prefrontal cortex (F3) Cathode: Right dorsolateral prefrontal cortex (F4)	1.5mA	20min	BICAMS, ANT,IIV
Simani et al. (22)	2022	Parallel	Iran	20	20	tDCS+CT	CT	17:3	18:2	Anode: Left dorsolateral prefrontal cortex (F3) Cathode: Right shoulder	2mA	30min	IVA-2
Hanken et al. (23)	2016	Parallel	Germany	20	20	tDCS+ vigilance task	shamtDCS+ vigilance task	13:7	12:8	Anode: Right parietal cortex (P4) Cathode: The contralateral forehead	1.5mA	20min	RT
Fiene et al. (24)	2018	Crossover	Germany	15		Each patient was randomized to receive 2 tDCS blocks with 1 week washout interval.		7:8		Anode: Left dorsolateral prefrontal cortex (F3) Cathode: Right shoulder	1.5mA	30min	RT
Palm et al. (25)	2016	Crossover	Germany	16		Each patient was randomized to receive 2 tRNS blocks, each consisting of 3 consecutive daily sessions with a 3-week washout interval.		13:3		Anode: Left dorsolateral prefrontal cortex (F3) Cathode: AF8	2mA	–	ANT
Chalah et al. (26)	2016	Crossover	France	10		Each patient was randomized to receive three blocks: Anodic tDCS for the left DLPFC, anodic tDCS for the right PPC, and two pseudotdcs for cortical sites		6:4		Anode: Left dorsolateral prefrontal cortex (F3) Cathode: located in the right supraorbital region Anode:the right parietal cortex (P4); Cathode Cz	2mA	20min	ANT
Ayache et al. (27)	2016	Crossover	France	16		Group 1: tDCS true stimulation followed by false stimulation	Group 2: tDCS false stimulation and then true stimulation	13:3		Anode: Left dorsolateral prefrontal cortex (F3) Cathode: Located in the right supraorbital region	2mA	20min	SDMT
Grigorescu et al. (28)	2020	Crossover	Germany	11		Patients were randomly assigned to receive either tDCS stimulation or sham stimulation with a 3-week washout interval between blocks		8:3		Anode: Left dorsolateral prefrontal cortex (F3) Cathode: Right dorsolateral prefrontal cortex (F4)	2mA	20min	SDMT
Mattioli et al. (29)	2015	Parallel	Italy	10	10	tDCS+CT	shamtDCS+CT	7:3	9:1	Anode: Left dorsolateral prefrontal cortex (F3) Cathode: Right shoulder	2mA	20min	SDMT

Exp, Experimental group; Ctrl, Control group; tDCS, Transcranial direct current stimulation; RS-tDCS, Remotely Supervised tDCS; TRNS, Transcranial random noise stimulation; Lf-rtms, Low frequency repeated transcranial magnetic stimulation; DLPFC, dorsolateral cortex; PPC, posterior parietal cortex; CT, Cognitive training; RT, Reaction Time; ANT, Attention network test; SDMT, Number symbol test; IIV, Individual variation index (response time).

**Table 2:** Included motor-related studies characteristics.

**Study**	**Year**	**Study design**	**Country**	**Sample Size**		**Interventions**		**Sex (Female: Male)**		**Intervention site**	**Stimulation intensity**	**Stimulation time**	**Outcome indicator**
				**Exp**	**Ctrl**	**Exp**	**Ctrl**	**Exp**	**Ctrl**				
Salemi et al. (30)	2019	Parallel	Italy	9	8	tRNS	shamtRNS	6:3	6:2	Anode: the entire motor cortex M1; Cathodel: The opposite cortex of the frontal lobe	1.5mA	15min	T25-FW
San et al. (31)	2019	Parallel	Turkey	10	6	rTMS+PT	shamrTMS +PT	4:6	4:2	The vertex region	110% of the resting motor-unit potential threshold	15min	MAS, PSFS,
Baroni et al. (32)	2022	Parallel	Italy	8	8	tDCS+TOCT	shamtDCS +TOCT	4:4	4:4	Anode: The right cerebellar cortex Cathode: The right buccinators muscle	2mA	15min	TUG, F8W,MSWS-12
Pilloni et al. (33)	2020	Parallel	Italy	9	8	tDCS+AE	shamtDCS +AE	-	-	Anode: Motor cortex M1 (C3) Cathode: Supraorbital outlet (Fp2)	2.5mA	20min	TUG time
Pilloni et al. (34)	2020	Parallel	Italy	9	6	tDCS+AE	shamtDCS +AE	6:3	5:1	Anode: Motor cortex M1 (C3) Cathode: Supraorbital outlet (Fp2)	2.5mA	20min	MSWS-12,
Darwish et al. (35)	2019	Parallel	Egypt	15	15	LF-rTMS stimulation combined with routine training	Routine training	10:5	8:07	The ipsilateral motor area M1 of the weaker limb	90% of the resting motor threshold	-	5STS,
Iodice et al. (36)	2015	Parallel	Italy	10	10	tDCS	shamtDCS	8:2	7:3	Anode: Weak side of motor cortex M1 Cathode: Weak side supraorbital outlet (Fp2)	2mA	20min	MSWS-12, MAS
Mori et al. (37)	2011	Parallel	Italy	10	10	iTBS+ET	shamiTBS+ET	3:7	4:6	the scalp site corresponding to the leg area of primary motor cortex contralateral to the affected limb.	80% of the active motor threshold	-	MAS

Exp, Experimental group; Ctrl, Control group; tDCS, Transcranial direct current stimulation; MAS, Modified Ashworth Scale; PSFS, Penn Spasm Frequency Scale; TUG, Timed Up and Go; T25WT, Timed 25 Foot Walking Test; 5STS, 5 repeated sit-standing tests; Msws-12, Multiple sclerosis Walking Scale.

**Table 3:** The characteristics of patients.

**Study**	**Year**	**Mean Age**		**Mean/Median EDSS**		**Course of disease**		**Subtypes**	
		**Exp**	**Ctrl**	**Exp**	**Ctrl**	**Exp**	**Ctrl**	**Exp**	**Ctrl**
**Cognition**									
Charvet et al. (21)	2018	52.69	51.00	-	-	17.71	15.70	7RR 18 OS	15RR 5 OS
Simani et al. (22)	2022	30.60	34.8	-	-	4.86	4.61	20RR	20RR
Hanken et al. (23)	2016	51.35	46.8	4.4	3.95	-	-	8RR 12CP	7RR 13CP
Fiene et al. (24)	2018	43.20		3.54		9.63		14RR 1OS	
Palm et al. (25)	2016	47.4		4.2		12.5		11RR 1SP 4PP	
Chalah et al. (26)	2016	40.50		2.3		14.0		9RR 1SP	
Ayache et al. (27)	2016	48.9		4.25		11.8		11RR 4SP 1PP	
Grigorescu et al. (28)	2020	43.91		3.14		75.64 Months		10RR 1SP	
Mattioli et al. (29)	2015	38.2	47.4	2.9	2.1	6.6	11.0	10RR	10RR
**Motor**									
Salemi et al. (30)	2018	39.8	44.2	2.8	2	–	–	–	–
San et al. (31)	2019	48.70	53.00	–	–	14.70	19.50	–	–
Baroni et al. (32)	2022	55.25	52.13	4.69	4.5	11.13	11.13	3RR 4PP 1SP	3RR 3PP 2SP
Pilloni et al.A (33)	2020	52.1	54.2	5.5	5	–	–	–	–
Pilloni et al.B (34)	2020	52.1	53.5	5.3	4.5	–	–	2RR 7SP	3RR 3SP
Darwish et al. (35)	2019	32.20	31.13	2	3	–	–	15RR	15RR
Iodice et al. (36)	2015	43.3	40.3	3.6	3.8	7.0	7.8	10RR	10RR
Mori et al. (37)	2011	39.1	37.7	3.6	3.8	–	–	–	–

Exp, Experimental group; Ctrl, Control group; EDSS, Extended Disability Status Scale; RR, Relapse-remitting multiple sclerosis; PP, Primary progressive multiple sclerosis; SP, Secondary progressive multiple sclerosis; CP, Chronic Progressive multiple sclerosis; OS, Other subtypes.

### 3.2. Interventions

Among the 17 included trial groups, two received rTMS (31, 35), two used tRNS (25, 30), one received remote tDCS (21), one applied iTBS (37) and the remaining 11 groups were all studied for the use of tDCS (22–24, 26–29, 32–34, 36). In total, nine trials (21, 23, 26–29, 33, 34, 36) set a single 20-min intervention, three trials (30–32) having a stimulus duration of 15 min for one intervention, and two trails (22, 24) applied a single 30-min intervention; this parameter was not described in three additional studies (25, 35, 37). Regarding the cognitive function (21, 25–30), all 10 investigations used a stimulus intensity of ≤2 mA, with a stimulation location in the left dorsolateral prefrontal cortex, except one study located over the right parietal cortex (P4) (23). For motor function, the stimulus intensity included 2.5, 2, 1.5 mA for TES; 110% of the resting motor-unit potential threshold, 90% of the resting motor threshold, and 80% of the active motor threshold for TMS. The stimulation site located over the motor cortex, right cerebellar cortex or the vertex region.

### 3.3. Outcome indicators

Different outcome indicators were used for the evaluation of different sectors in the included studies. The evaluation indicators of cognition included the Attention Network Test (ANT), Symbol Digit Modalities Test (SMDT), reaction time (RT) and intra-individual variability (IIV); the 12-item Multiple Sclerosis Walking Scale (MSWS-12), 5-Repetition Sit-to-Stand Test (5STS), Penn Spasm Frequency Scale (PSFS), Timed Up and Go (TUG), Modified Ashworth Scale (MAS), and Timed 25 Foot Walking Test (T25FWT) were applied in motor function related research.

### 3.4. Research quality

**Figure 2:**
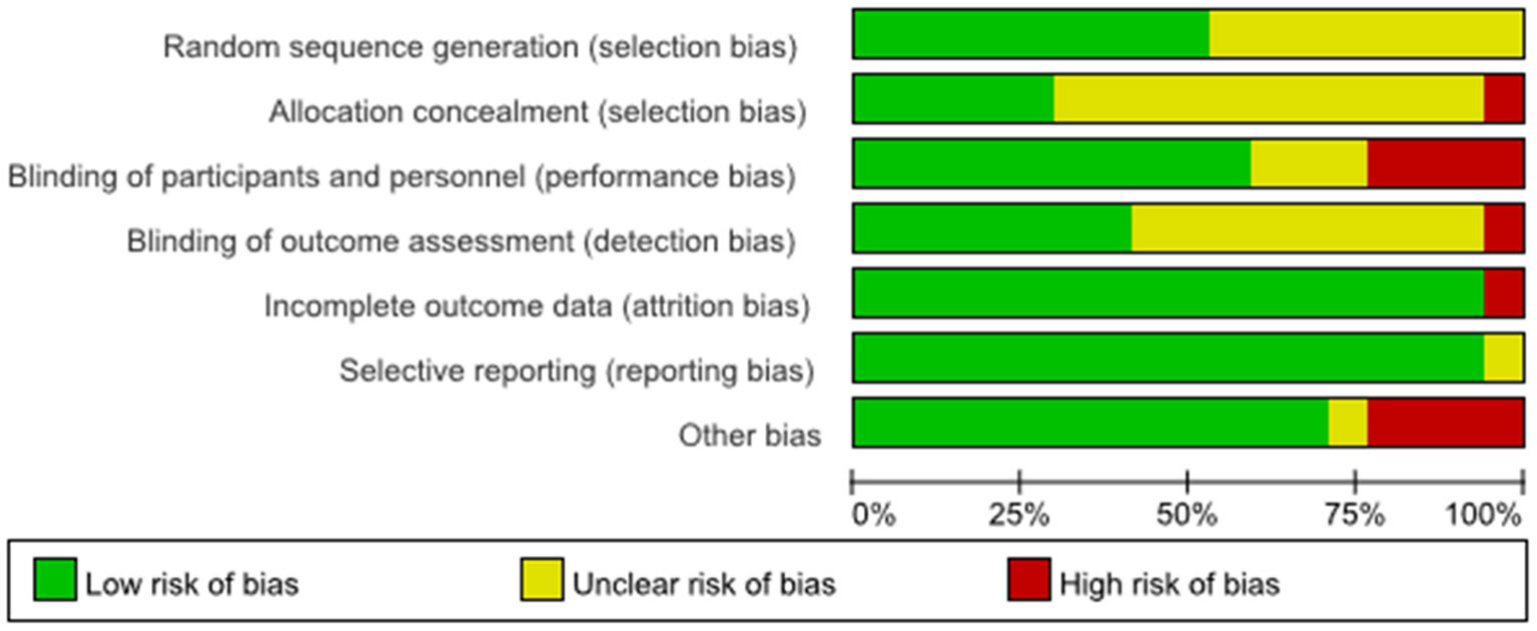
Risk of bias graph.

**Figure 3:**
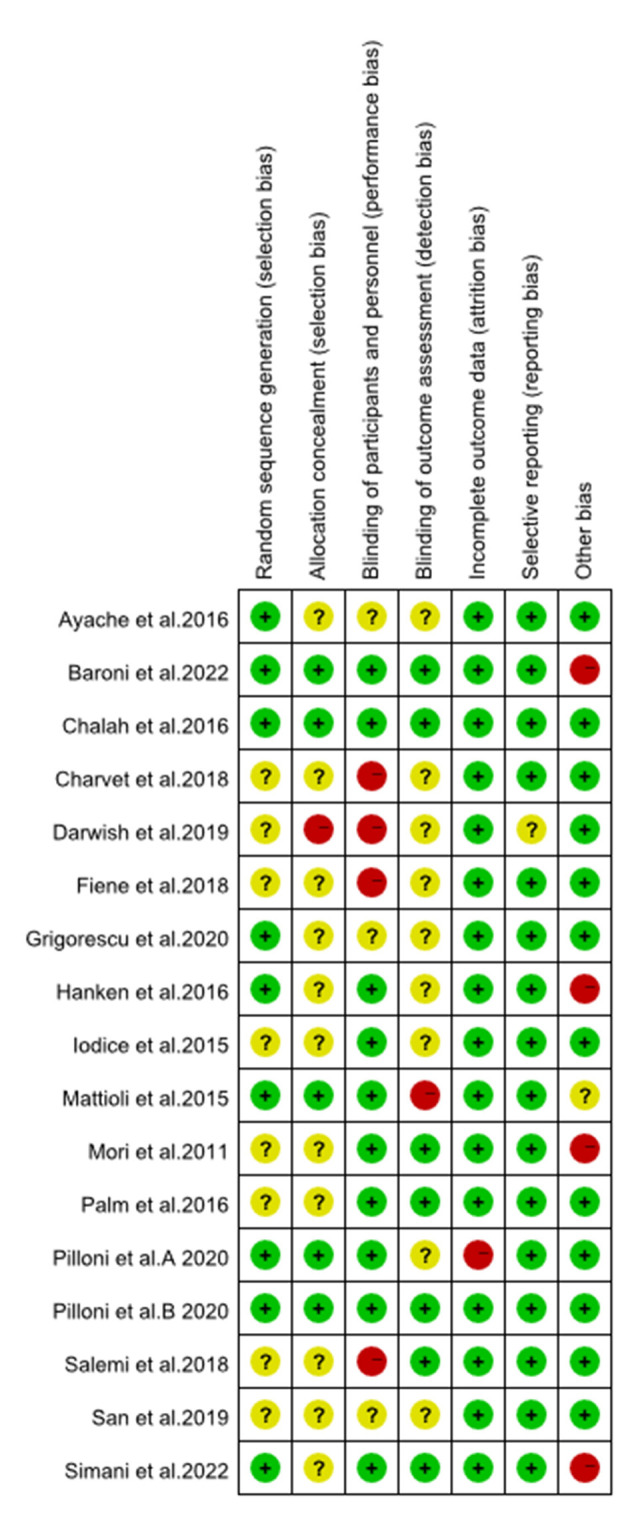
Risk of bias summary.

The results of the evaluation of the quality of the included articles showed in Figures 2, 3, the randomization method was not reported in five of them (21, 25, 30, 35, 36), and the methods reported in the remaining articles included computer randomization, technician randomization, and randomization list. Five articles (26, 29, 32–34) reported its allocation and concealment; most of the studies included were double-blinded and apart from that, three studies (27, 28, 31) didn't reported its blind method, two articles (21, 35) didn't use blind method, and two articles (24, 30) used single blind method. Six studies (25–27, 29, 30, 32) reported adverse effects, such as headache, nausea, itches, and insomnia.

### 3.5. Meta-analysis

The study topic, outcome measures, number of participants, and mean and standard deviation of the post-treatment evaluation for each trial were summarized in the Supplementary material. The Supplementary material contains all the codes used for STATA software analysis, and also the results of software calculations as well.

#### 3.5.1. Cognitive function

**Figure 4:**
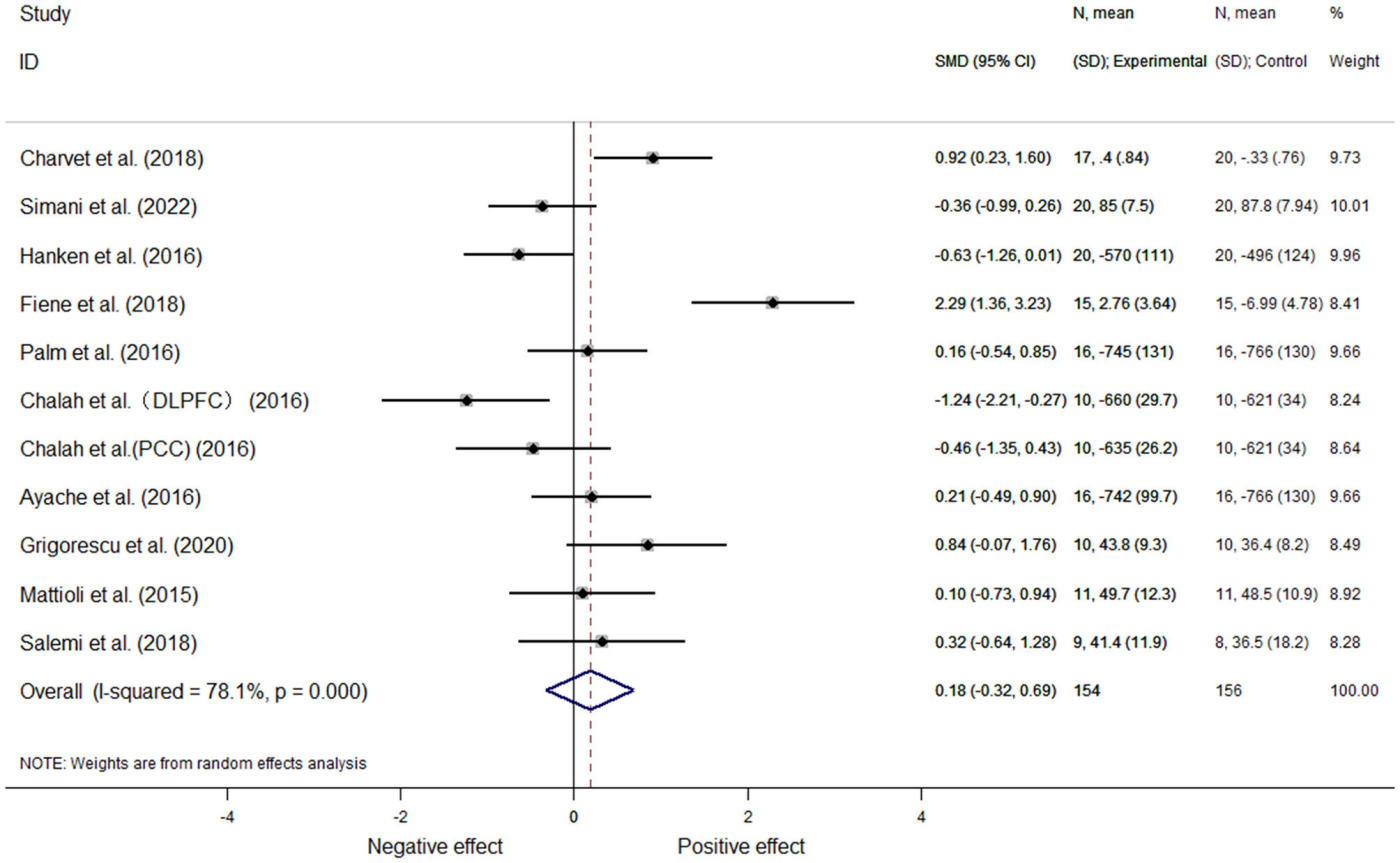
Cognitive function.

Based on a total of ten articles, 11 effect sizes were calculated. In one article (26), two sets of data could be extracted according to the stimulation site. Because the included study reported different outcome measures, we used the mean standard deviation to represent the effect size; moreover, for the analysis of heterogeneity, we utilized a random-effects meta-analysis (*I*^2^ = 78.1%, *P* = 0.00). This analysis revealed there was no significant differences [SMD = 0.18, 95% CI (−0.32, 0.69), *P* = 0.475] (Figure 4).

##### 3.5.1.1. Subgroup analysis based on treatment modality

We performed subgroup analyses of cognition-related studies separately based on different outcome indicators (IVV, FSAQ, RT, ANT, and SDMT), interventions (tDCS and tRNS), treatment duration (15, 20, and 30min), treatment intensity (1.5 and 2mA), and stimulation site (F3 and P4) reported, and due to heterogeneity among studies in each categorical subgroup above, random-effects models were used for analysis. The results showed that the NIBS located on P4 had a negative effect on cognition function, with a statistically significant difference [SMD = 0.41, 95% CI (0.09, 0.73), *P* = 0.01]. No statistically significant differences were found between rest studies in subgroups with two or more studies. The Subgroup data, analysis results, and forest plots are available in the Supplementary material 3.1.1.

##### 3.5.1.2. Subgroup analysis based on patient characteristics

Subgroup analysis was performed separately in terms of mean age (age >45 and age < 45) and mean EDSS (EDSS >3.5 and EDSS < 3.5) of study subjects. A random-effects model was used due to heterogeneity between studies within each subgroup, and the analysis found no statistically significant differences between studies in subgroups containing two or more studies (Supplementary material 3.1.2).

##### 3.5.1.3. Analysis of the attention network test

**Figure 5:**
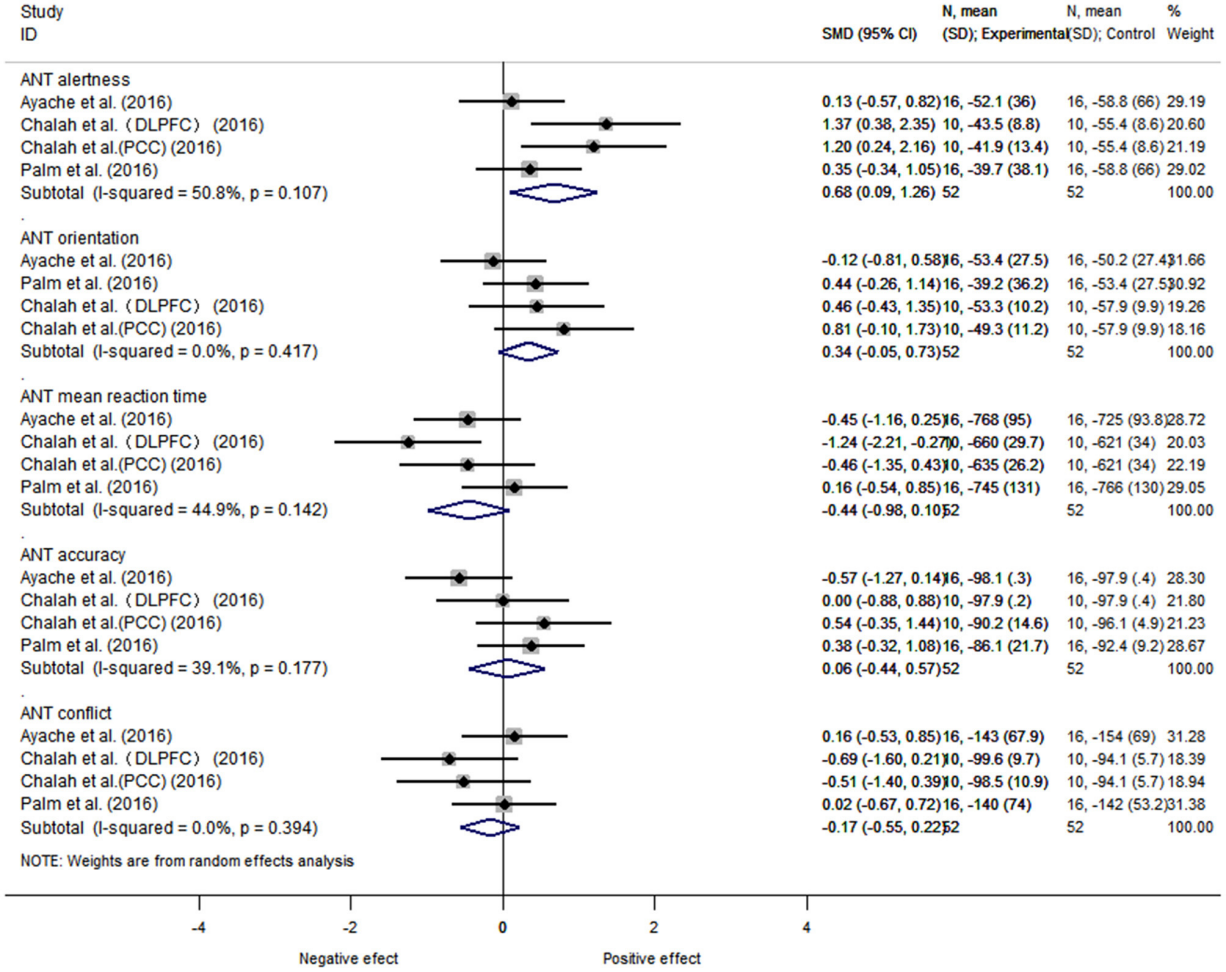
ANT subgroup.

Further grouping according to the results of 5 items of the ANT test, the data of 3 articles, 5 items, and 42 subjects was performed. There was heterogeneity between alertness subgroup (*I*^2^ = 50.8%, *P* = 0.18), and a fixed-effects model was used. The heterogeneity between orientation (*I*^2^ = 0%, *P* = 0.00), execution/conflict (*I*^2^ = 0%, *P* = 0.00), average reaction time (*I*^2^ = 44.9%, *P* = 0.16), and average accuracy (*I*^2^ = 39.1%, *P* = 0.10) were considered as small. We found that the NIBS intervention had a better effect on improving alertness compared with the control group, with a statistically significant difference [SMD = 0.68, 95% CI (0.09, 1.26), *P* = 0.02]. There was no statistically significant difference between the rest groups (orientation [SMD = 0.34, 95% CI (−0.05, 0.73), *P* = 0.09], execution/conflict [SMD = −0.17, 95% CI (−0.55, 0.23), *P* = 0.40], average reaction time [SMD = −0.44, 95% CI (−0.98, 0.10), *P* = 0.11], and average accuracy [SMD = 0.06, 95% CI (−0.44, 0.57), *P* = 0.80]) (Figure 5).

#### 3.5.2. Motor function

**Figure 6:**
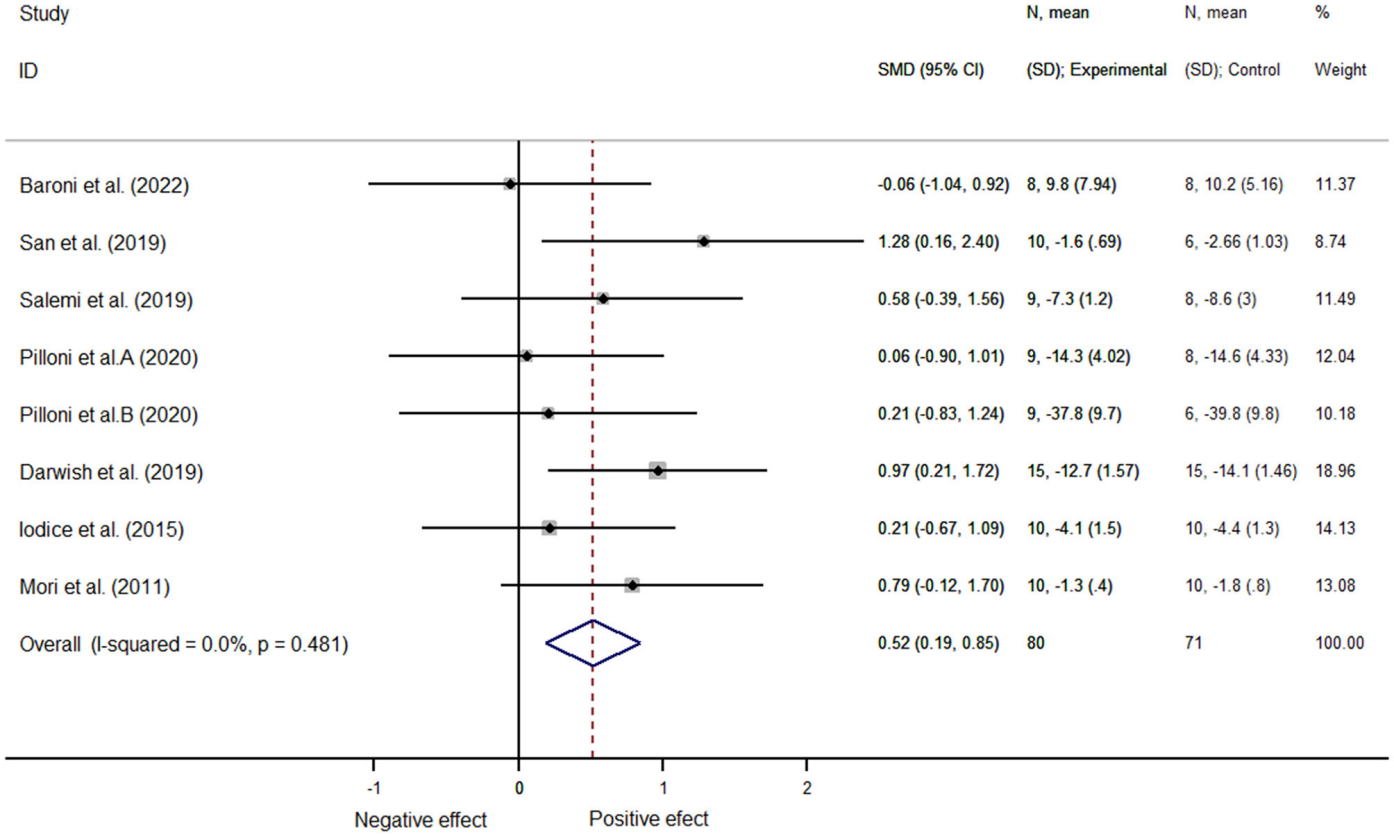
Motor function.

In total, 151 patients from eight articles were analyzed. Similarly, since the included studies included multiple outcome indicators, we used mean standard deviation to represent the effect size, and computer software analysis showed no heterogeneity (*I*^2^ = 0.0%, *P* = 0.48); we therefore selected the fixed-effects model for data analysis and found that the NIBS intervention improved motor function better than did the control one, with a statistically significant difference [SMD = 0.52, 95% CI (0.19, 0.85), *P* = 0.002] (Figure 6).

##### 3.5.2.1. Subgroup analysis based on treatment modality

We also performed subgroup analyses of motor-related studies, respectively, based on different outcome indicators (MSWS12, MAS/PSFS, T25FWT, TUG, and 5STS), interventions (TES and TMS), treatment duration (15 and 20min), treatment intensity (1.5, 2, and 2.5mA), and stimulation site (right cerebellar cortex, lower extremity motor area of cerebral cortex, and motor cortex M1) reported. Each of the subgroups had no study heterogeneity, and a fixed-effects model was used for analysis. The analysis revealed statistically significant differences between subgroup studies with a MAS/PSFS outcome indicator [SMD = 0.68, 95% CI (0.13, 1.23), *P* = 0.015], a TMS intervention [SMD = 0.98, 95% CI (0.46, 1.49), *P* = 0.000], a motor cortex M1 location [SMD = 0.52, 95% CI (0.15, 0.88), *P* = 0.006], all with a positive effect. There was no statistically significant difference between the rest groups (Supplementary material 3.2.1).

##### 3.5.2.2. Subgroup analysis based on patient characteristics

Subgroup analysis was performed separately in terms of mean age (age > 45 and age < 45) and mean EDSS (EDSS > 3.5 and EDSS < 3.5) of motor-related study subjects. Furthermore, there was no heterogeneity between studies within each subgroup, and a fixed-effects model was applied. The differences between studies in subgroups with age < 45 [SMD = 0.67, 95% CI (0.23, 1.10), *P* = 0.003] and EDSS < 3.5 [SMD = 0.82, 95% CI (0.22, 1.42), *P* = 0.007] were statistically significant, and all had positive effect (Supplementary material 3.2.2).

### 3.6. Meta-regression analyses

Meta-regression analyses were conducted to investigate the patients' factors influencing the treatment effect. For cognition function, mean age (*P* = 0.525) and EDSS (*P* = 0.726) did not influence the treatment effect, accorded with the associated subgroup analyses results in our study; For motor function, mean age (*P* = 0.166) and EDSS (*P* = 0.143) did not influence the treatment effect as well, which was odd with the associated subgroup analyses results (Supplementary material 3.3).

### 3.7. Publication bias and sensitivity analysis

The number of articles included in this study was < 10 for each topic; therefore, a funnel plot was not used for publication bias analysis. The sensitivity analysis performed here consisted of sequentially removing the studies one by one and, at each step, performing a meta-analysis of the remaining studies. The results were compared to those obtained before the previous step. We didn't found significant change when only remove an article at a time but it should be noted that the heterogeneity decreased from 78.1 to 6.7% after four trails were excluded from the overall cognitive function analysis (21, 23, 24, 26), suggesting that these article may have been the source of the heterogeneity detected between the cognitive function analysis groups and the process was shown in the Supplementary material 3.4.

## 4. Discussion

In total, 17 articles written in English were included in this study, wherein 364 patients were examined. Our findings imply that although no significant deference that NIBS improves cognitive function was found, it might help pwMS patients with their motor function. Further subgroup analysis revealed findings suggesting that stimulation located on P4 did not improve patient cognition, as well as that TES may improve cognitive alertness in pwMS. In particular, NIBS contributed to the improvement of MAS/ PSFS scores in pwMS, while TMS as an intervention, stimulation of area M1 was found to possibly improve motor function in pwMS. Additionally, patients with EDSS scores under 3.5 and those under the age of 45 experienced a therapeutic benefit from NIBS according to the results.

We didn't found evidence to prove that NIBS can improve the cognitive function of pwMS and the differences between the studies were not statistically significant for the different stimulus intensities, single stimulus durations, outcome indicators, and patient characteristics (EDSS and Age). At present, the mechanism *via* which NIBS affects cognitive function in pwMS remains controversial; however, a possible mechanism consists in the modulation of patient function by modulating cortical excitability (41) or by affecting neuroplasticity (42). Among the non-invasive brain stimulation models in cognitive neuroscience, there are differences in the mechanisms *via* which the various types of NIBS can affect neurological function, such as the induction of the suprathreshold depolarization of neurons by TMS and the induction of the subthreshold polarization of neurons in the stimulated area by transcranial electric stimulation (tES) (43). In particular, the cognitive-related studies in our study only used tDCS or tRNS., the findings are only currently applicable to tDCS and tRNS in terms of NIBS intervention and require the generation of additional experimental data for validation and in-depth analysis.

It is also important to note the large heterogeneity among the included cognitive-related studies (*I*^2^ > 75%), and sensitivity analysis found four articles (21, 23, 24, 26) with a large effect on heterogeneity. In terms of patients' characteristics, the mean age of Charvet et al.'s study was the oldest and its mean disease duration was deemed as the longest. Furthermore, the study of Chalah et al. had a 6:4 male-to-female ratio, which is inconsistent with MS onset characteristics; in terms of interventions, the study of Chalah et al. and Hanken et al. were the only experiments that acted on the right parietal cortex (P4); In terms of study design, neither Charvet et al. nor Fiene et al. used a double-blind design, and randomization methods and allocation concealment were not reported; these all could be the reasons for heterogeneity.

The ANT, developed to measure the function of the attentional networks (alertness, orientation, and execution/conflict) is based on a complex computer system and includes a total of five cognitive-related outcome indicators, two of which are generalized (mean reaction time and mean accuracy) (44, 45). The ANT parameters include five sections, and a subgroup analysis showed that alertness function was significantly improved. This may because alerting network is linked to thalamic and frontoparietal areas of the left hemisphere (46) and 3 trails among a total of 4 stimulated the left DLPFC. In addition, one study showed that attentional deficits were a “cognitive feature” of MS fatigue (23), and Chalah et al. (26) showed that left DLPFC helped to improve patients' fatigue and therefore attentional performance. Similarly, the site P4 helps to reduce task-related reaction time and the effect was influenced by the level of fatigue (23). Notably, tDCS over the right DLPFC also helps increase attention (47).

NIBS can improve the motor function of pwMS. Moreover, because the primary study indicators included in this study focused on lower-extremity function, this finding is more applicable to lower-extremity motor function. Early symptoms of MS include weakness in one or more limbs, with lower limb motor dysfunction being more severe than upper limb motor dysfunction (4, 48).

There was an improvement in outcome indicators for spasticity (MAS/ PSFS), a common symptom of MS and an important motor disorder that causes walking difficulties and even disability in pwMS (49, 50). This subgroup contained three trails, and to our knowledge, the study of Iodice et al. (36) is the only study examining the effect of tDCS on spasticity in MS patients, did not find significant improvement, contrary to the results of the other two TMS studies. More studies on the application of tDCS are needed to further confirm this result. TMS as an intervention or stimulation site on M1 can improve motor function and these findings are consistent with previous study (51). The promising results reported for the M1 region may because this region has multiple connections between the brain and peripheral areas and its effectiveness in treating spasticity have been reported (51). Moreover, a guideline has suggested for MS there is probable efficacy of iTBS of the leg area of M1 contralateral to the most affected limb (or both M1) in lower limb spasticity (Level B) (52).

We observed an improvement in motor function in patients under 45 years and patients with an EDSS score < 3.5 after the NIBS intervention in the subgroup analysis. EDSS is the most widely used disability and impairment rating scale in MS (53). With a total score of 10, the higher EDSS score stands for the more severe disability condition and 3.5 is the condition of fully ambulatory but with moderate disability in one function system (54). The regression analysis of these two characteristics of patients found that the trend of the regression line was consisted with the subgroup analysis result, but the regression did not achieve statistical significance. What should be noted is this does not mean that NIBS only improves motor function in the group of patients younger than 45 years or with an EDSS <3.5. This result may suggest that motor improvement is more pronounced in younger and less severely affected patients and leads us to recommend early NIBS intervention in clinical pwMS patients to achieve better intervention outcomes. The cut-off points were set based on data from the included studies, and additional studies would help to further validate this speculation as well as find more precise cut-off points. Furthermore, MS primarily affects people between the ages of 20 and 40 years. According to the findings of this study, most pwMS can benefit from NIBS to alleviate their motor dysfunction.

Previous evidence-based medical analyses have examined the effects of NIBS on functional problems related to pain, overall cognition, and fatigue in pwMS. To the best of our knowledge, no studies have analyzed motor and specific cognitive domains (attention); thus, this study reports the first meta-analysis aimed at exploring the effects of NIBS on attentional vigilance and orientation functions in pwMS. Our study included the broad category of non-invasive brain stimulation, which may serve as a reference for the use of NIBS techniques other than pharmacotherapy. Given that the majority of the research material focused on generic NIBS procedures, such as tDCS and rTMS, this study included therapies beyond those procedures, which has a certain reference value for the various choices of NIBS. In addition, we included a trial of remote tDCS techniques in this study, to provide some guidance regarding their different applications.

This paper explored the effects of NIBS on the cognitive and motor functions of pwMS; however, some limitations of this study should be pointed out. First, the number of trials included was not sufficient to further investigate the application of the NIBS technology and due to this biggest limitation, this article does not explore combined use of NIBS, in combination with traditional cognitive training, NIBS can be used to enhance the forms of neuroplasticity that facilitate functional recovery (55). Munoz-Paredes et al. (56) found that by participating in an exercise program and receiving tDCS separately pwMS underwent a positive impact. Tramontano et al. (57) reported that combined cerebellar iTBS and VR improves gait and balance abilities more than standard VR treatment in pwMS. In light of these findings, we suggest future studies to explore combined use of NIBS, including but not limited to combining other training (concurrently or separately), the use of multiple sites, etc. Then, the findings of our study are limited in some aspect, since the application of TMS was not included, the results of the cognitive function analysis were only applicable to tES; no distinction was made between MS subtypes, and only show the short-term effect of NIBS, limiting the findings to some extent. Third, English literature alone was included in the meta-analysis, which could have resulted in a linguistic bias. Moreover, this study indicated that the attention of pwMS in other areas needs to be improved urgently. Other constraints included the source, depth, and quality of the existing evidence, all of which are common in meta-analysis.

In conclusion, the results of this present study provide evidence that non-invasive brain stimulation could improve alertness in pwMS, but it did not improve overall cognitive function in pwMS. Furthermore, NIBS may help pwMS with motor function and those who are under 45 years of age or with EDSS < 3.5 improve their motor function. According to our findings, for the therapeutic use of NIBS, the recommended intervention modality is TMS as an intervention and located on the M1 and we also suggest early intervention to obtain more improvement. Because of the limited sample size, the conclusion of this study still needs to be verified in additional studies. We hope that this study will encourage researchers to pay more attention to pwMS and conduct relevant studies on the application of the NIBS technology in MS, to improve the quality of life of this population.

## Data availability statement

The original contributions presented in the study are included in the article/Supplementary material, further inquiries can be directed to the corresponding authors.

## Author contributions

XZ, JZ, and QZeng: study conception and design. YZ, LC, and GL: literature search, studies selection, and data collection. ShuL, QZhang, and SZ: analysis and interpretation of data, preparing figures, and editing the draft. ShiL, LH, and SC: study supervision and reviewing of the final manuscript. All authors contributed to the article and approved the submitted version.
