# Supplementary material for: Efficacy of non-invasive brain stimulation on cognitive and motor functions in multiple sclerosis: A systematic review and meta-analysis
Source: Front Neurol. 2023 Jan 26;14:1091252. doi: 10.3389/fneur.2023.1091252 (PMC9911042; doi:10.3389/fneur.2023.1091252)
Supplement: Supplementary file 1 [file Data_Sheet_1.PDF]

frontiers

Efficacy of Non-invasive Brain Stimulation on Cognitive and Motor Functions in Multiple Sclerosis: A systematic review andmeta-analysis

1 Search strategy 2
1.1 Pubmed 2
1.2 CNKI 2
2 Data for analysis 3
3 Software results 7
3.1 Cognition function 7
3.1.1 Subgroup Analysis Based on Treatment Modality 8
3.1.1.1 Outcome indicators 8
3.1.1.2 Interventions 10
3.1.1.3 Intensity 12
3.1.1.4 Duration 14
3.1.1.5 Stimulation site 16
3.1.2 Subgroup Analysis Based on Patient Characteristics 18
3.1.2.1 Mean age 18
3.1.2.2 Mean EDSS 20
3.1.3 Analysis of the Attention Network Test 22
3.2 Motor function 24
3.2.1 Subgroup Analysis Based on Treatment Modality 25
3.2.1.1 Outcome indicators 25
3.2.1.2 Interventions 27
3.2.1.3 Intensity 29
3.2.1.4 Duration 31
3.2.1.5 Stimulation site 33
3.2.2 Subgroup Analysis Based on Patient Characteristics 34
3.2.2.1 Mean age 34
3.2.2.2 Mean EDSS 36
3.3 Meta regression 38
3.3.1.1 Mean Age of Cognition 38
3.3.1.2 Mean EDSS of Cognition 39
3.3.1.3 Mean Age of Motor 40
3.3.1.4 Mean EDSS of Motor 41
3.4 sensitivity analysis of cognitive-related studies 41
3.4.1 Remove Fiene et al. 42
3.4.2 Remove Chalah et al.（DLPFC） 43
3.4.3 Remove Charvet et al. (2018） 44
3.4.4 Remove Hanken et al. 44

1 Search strategy

1.1 Pubmed

((((((((((((((((((((((Cognitive Impairments) OR (cognition disorder)) OR (Cognition)) OR (Cognitions)) OR (Cognitive Function)) OR (Cognitive Functions)) OR (Motor Dysfunction)) OR (motor function)) OR (Cognitive Dysfunction)) OR (Processing Speed)) OR (Working Memory)) OR (Attention)) OR (Vigilance)) OR (Social Cognition)) OR (Executive Functioning)) OR (Muscle Spasticity)) OR (Spasm)) OR (Mobility Limitation)) OR (Ambulation Difficulty)) OR (Difficulty Walking)) OR (Gait)) AND ((((((((((((((((((((((Transcranial Direct Current Stimulation) OR (Cathodal Stimulation Transcranial Direct Current Stimulation)) OR (Cathodal Stimulation tDCS)) OR (Cathodal Stimulation tDCSs)) OR (Transcranial Random Noise Stimulation)) OR (Transcranial Alternating Current Stimulation)) OR (Transcranial Electrical Stimulation)) OR (Transcranial Electrical Stimulations)) OR (Anodal Stimulation Transcranial Direct Current Stimulation)) OR (Anodal Stimulation tDCSs)) OR (Anodal Stimulation tDCS)) OR (Repetitive Transcranial Electrical Stimulation)) OR (Transcranial Magnetic Stimulation)) OR (pulsed electromagnetic field therapy)) OR (low field magnetic stimulation)) OR (Noninvasive brain stimulation)) OR (Non-invasive brain stimulation)) OR (NIBS)) OR (Repetitive Transcranial Magnetic Stimulation)) OR (Single Pulse Transcranial Magnetic Stimulation)) OR (Paired Pulse Transcranial Magnetic Stimulation)) OR (theta burst stimulation))) AND ((((("Multiple Sclerosis"[Mesh]) OR "Multiple Sclerosis, Chronic Progressive"[Mesh]) OR "Multiple Sclerosis, Relapsing-Remitting"[Mesh]) OR (MS[Title/Abstract])) OR (Disseminated Sclerosis[Title/Abstract]))

Filters applied: Clinical Trial

1.2 CNKI

(非侵入性脑刺激 + 经颅电刺激 + 经颅磁刺激) AND (多发性硬化 + 多发性硬化症 + MS) AND (认知 + 运动 + 痉挛状态 + 肌肉痉挛)

2 Data for analysis

Table 1 Data for cognition

Study	Year	Outcomes	T_E	m_E	SD_E	T_C	m_C	SD_C	Intervention	Intensity	Time	EDSS	Age	Mean age	Mean EDSS	Location
Charvet et al.	2018	IIV	17	0.4	0.84	20	-0.33	0.76	tDCS	1.5mA	20min		51.9389	age >45		Left dorsolateral prefrontal cortex (F3)
Simani et al.	2022	FSAQ	20	85.04	7.5	20	87.84	7.94	tDCS	2mA	30min		32.7	age <45		Left dorsolateral prefrontal cortex (F3)
Hanken et al.	2016	RT	20	-570.35	110.73	20	-496.45	123.99	tDCS	1.5mA	20min	4.175	49.075	age >45	EDSS >3.5	Right parietal cortex (P4)
Fiene et al.	2018	RT	15	2.76	3.64	15	-6.99	4.78	tDCS	1.5mA	30min	3.54	43.2	age <45	EDSS >3.5	Left dorsolateral prefrontal cortex (F3)
Palm et al.	2016	ANT	16	-745.3	131.4	16	-766.1	130.3	tRNS	2mA		4.2	47.4	age >45	EDSS >3.5	Left dorsolateral prefrontal cortex (F3)
Chalah et al.（DLPFC）	2016	ANT	10	-660.2	29.7	10	-620.6	34	tDCS	2mA	20min	2.3	40.5	age <45	EDSS <3.5	Left dorsolateral prefrontal cortex (F3)
Chalah et al.(PCC)	2016	ANT	10	-634.7	26.2	10	-620.6	34	tDCS	2mA	20min					Right parietal cortex (P4)
Ayache et al.	2016	ANT	16	-742.3	99.7	16	-766.1	130.3	tDCS	2mA	20min	4.25	48.9	age >45	EDSS >3.5	Left dorsolateral prefrontal cortex (F3)

Grigorescu et al.	2020	SDMT	10	43.8	9.3	10	36.4	8.2	tDCS	2mA	20min	3.14	43.91	age <45	EDSS <3.5	Left dorsolateral prefrontal cortex (F3)
Mattioli et al.	2015	SDMT	11	49.73	12.28	11	48.55	10.89	tDCS	2mA	20min	2.5	42.8	age <45	EDSS <3.5	Left dorsolateral prefrontal cortex (F3)
Salemi et al.	2018	SDMT	9	41.4	11.9	8	36.5	18.2	tRNS	1.5mA	15min	2.4	41.8706	age <45	EDSS <3.5	Left dorsolateral prefrontal cortex (F3)

T_E: total number of experimental group; m_E: mean of experimental group; SD_E: standard deviation of experimental group; T_C: total number of control group; m_C: mean of control group; SD_C: standard deviation of control group; EDSS: Extended Disability Status Scale; tDCS: Transcranial direct current stimulation; RS-tDCS: Remotely Supervised tDCS; TRNS: Transcranial random noise stimulation;Lf-rtms: Low frequency repeated transcranial magnetic stimulation;DLPFC: dorsolateral cortex;PPC: posterior parietal cortex;CT: Cognitive training; RT: Reaction Time; ANT: Attention network test;SDMT: Number symbol test;IIV: Individual variation index (response time)

Table 2 Data for motor

Study	Year	Outcomes	T_E	m_E	SD_E	T_C	m_C	SD_C	Intervention	Intensity	Time	EDSS	Age	Mean age	Mean EDSS	location
Baroni et al.	2022	MSWS12	8	9.8	7.94	8	10.19	5.16	TES	2mA	15min	4.6	53.69	age >45	EDSS >3.5	right cerebellar cortex
Şan et al.	2019	PSFS	10	-1.6	0.69	6	-2.66	1.03	TMS		15min		50.31	age >45		lower extremity motor area of cerebral cortex
Salemi et al.	2019	T25FWT	9	-7.3	1.2	8	-8.6	3	TES	1.5mA	15min	2.42	41.87	age <45	EDSS <3.5	motor cortex M1
Pilloni et al.A	2020	TUG time	9	-14.34	4.02	8	-14.58	4.33	TES	2.5mA	20min	5.26	53.09	age >45	EDSS >3.5	motor cortex M1
Pilloni et al.B	2020	MSWS12	9	-37.8	9.7	6	-39.8	9.8	TES	2.5mA	20min	4.98	52.66	age >45	EDSS >3.5	motor cortex M1
Darwish	2019	5STS	15	-12.6	1.56	15	-14.1	1.45	TMS			2.5	31.6	age <45	EDSS	motor cortex M1

et al.				7	6		3	7					65		<3.5	
Iodice et al.	2015	MAS	10	-4.1	1.5	10	-4.4	1.3	TES	2mA	20min	3.7	41.8	age <45	EDSS >3.5	motor cortex M1
Mori et al.	2011	MAS	10	-1.3	0.4	10	-1.8	0.8	TMS			3.7	38.4	age <45	EDSS >3.5	motor cortex M1

T_E: total number of experimental group; m_E: mean of experimental group; SD_E: standard deviation of experimental group; T_C: total number of control group; m_C: mean of control group; SD_C: standard deviation of control group; EDSS: Extended Disability Status Scale; tDCS: Transcranial direct current stimulation; MAS: Modified Ashworth Scale; PSFS: Penn Spasm Frequency Scale; TUG: Timed Up and Go; T25WT: Timed 25 Foot Walking Test; 5STS: 5 repeated sit-standing tests; Msws-12: Multiple sclerosis Walking Scale

Table 3 ANT subgroup

Study	Year	Outcomes	T_E	m_E	SD_E	T_C	m_C	SD_C
Ayache et al.	2016	ANT alertness	16	-52.1	36	16	-58.8	66
Chalah et al.（DLPFC）	2016	ANT alertness	10	-43.5	8.8	10	-55.4	8.6
Chalah et al.(PCC)	2016	ANT alertness	10	-41.9	13.4	10	-55.4	8.6
Palm et al.	2016	ANT alertness	16	-39.7	38.1	16	-58.8	66
Ayache et al.	2016	ANT orientation	16	-53.4	27.5	16	-50.2	27.4
Palm et al.	2016	ANT orientation	16	-39.2	36.2	16	-53.4	27.5
Chalah et al.（DLPFC）	2016	ANT orientation	10	-53.3	10.2	10	-57.9	9.9
Chalah et al.(PCC)	2016	ANT orientation	10	-49.3	11.2	10	-57.9	9.9
Ayache et al.	2016	ANT mean reaction time	16	-768.3	95	16	-725.4	93.8
Chalah et al.（DLPFC）	2016	ANT mean reaction time	10	-660.2	29.7	10	-620.6	34
Chalah et al.(PCC)	2016	ANT mean reaction time	10	-634.7	26.2	10	-620.6	34
Palm et al.	2016	ANT mean reaction time	16	-745.3	131.4	16	-766.1	130.3
Ayache et al.	2016	ANT accuracy	16	-98.1	0.3	16	-97.9	0.4
Chalah et al.（DLPFC）	2016	ANT accuracy	10	-97.9	0.2	10	-97.9	0.4

Chalah et al.(PCC)	2016	ANT accuracy	10	-90.2	14.6	10	-96.1	4.9
Palm et al.	2016	ANT accuracy	16	-86.1	21.7	16	-92.4	9.2
Ayache et al.	2016	ANT conflict	16	-143	67.9	16	-153.9	69
Chalah et al.（DLPFC）	2016	ANT conflict	10	-99.6	9.7	10	-94.1	5.7
Chalah et al.(PCC)	2016	ANT conflict	10	-98.5	10.9	10	-94.1	5.7
Palm et al.	2016	ANT conflict	16	-140.3	74	16	-141.8	53.2

T_E: total number of experimental group; m_E: mean of experimental group; SD_E: standard deviation of experimental group; T_C: total number of control group; m_C: mean of control group; SD_C: standard deviation of control group

3 Software results

3.1 Cognition function

. metan T_E m_E SD_E T_C m_C SD_C, cohen random label(namevar= Study , yearvar= Year) counts group1(Experimental) group2(Control) texts(> 220) xlabel(-4,-2,0,2,4)

Study	SMD	[95% Conf. Interval]		% Weight
Charvet et al. (2018	0.915	0.234	1.596	9.73
Simani et al. (2022)	-0.363	-0.988	0.263	10.01
Hanken et al. (2016)	-0.629	-1.264	0.007	9.96
Fiene et al. (2018)	2.295	1.360	3.230	8.41
Palm et al. (2016)	0.159	-0.535	0.853	9.66
Chalah et al.（DLPFC	-1.241	-2.206	-0.275	8.24
Chalah et al.(PCC) (	-0.465	-1.354	0.425	8.64
Ayache et al. (2016)	0.205	-0.490	0.900	9.66
Grigorescu et al. (2	0.844	-0.075	1.763	8.49
Mattioli et al. (201	0.102	-0.735	0.938	8.92
Salemi et al. (2018)	0.323	-0.636	1.282	8.28
D+L pooled SMD	0.183	-0.320	0.686	100.00

Heterogeneity chi-squared = 45.56 (d.f. = 10) p = 0.000

I-squared (variation in SMD attributable to heterogeneity) = 78.1%

Estimate of between-study variance Tau-squared = 0.5560

Test of SMD=0 : z= 0.71 p = 0.475

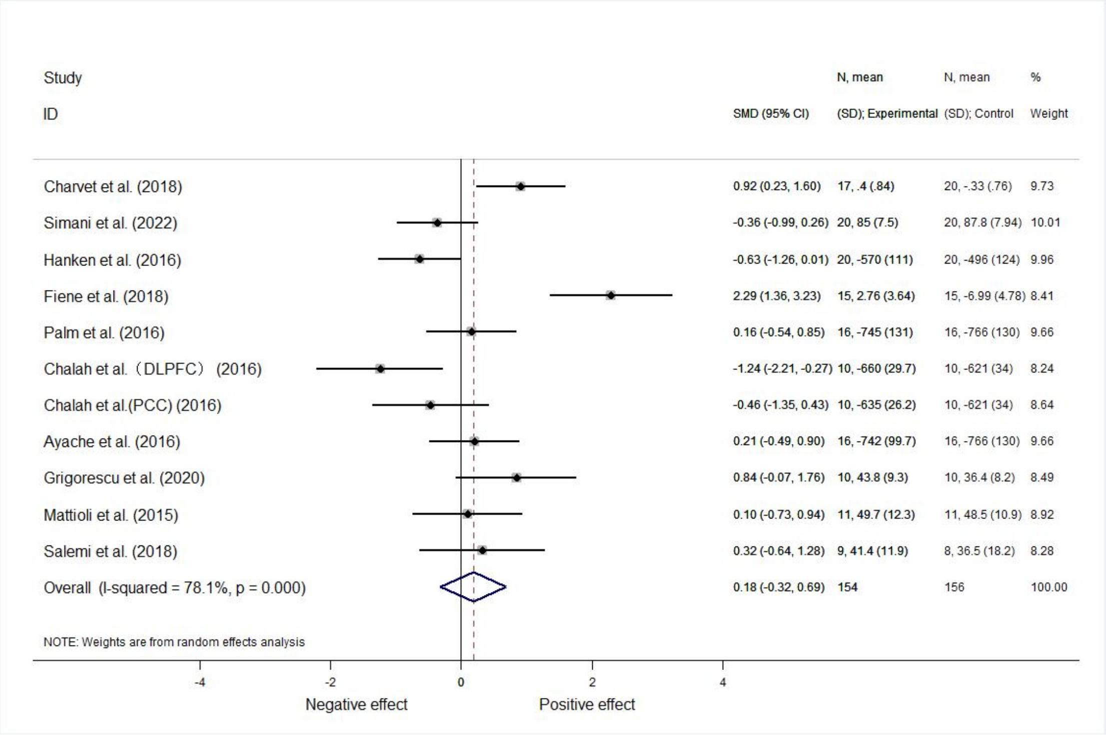

3.1.1 Subgroup Analysis Based on Treatment Modality

3.1.1.1 Outcome indicators

. metan T_E m_E SD_E T_C m_C SD_C, cohen random label(namevar= Study , yearvar= Year) counts group1(Experimental) group2(Control) texts(> 150) xlabel(-4,-2,0,2,4) by(Outcomes)

```
              Study         |       SMD    [95% Conf. Interval]       % Weight
---------------------+--------------------------------------------------
        IVV
Charvet et al. (2018 |      0.915        0.234        1.596                 9.73
  Sub-total                 |
    D+L pooled SMD          |      0.915        0.234        1.596                 9.73
---------------------+--------------------------------------------------
        FSAQ
Simani et al. (2022) |     -0.363       -0.988        0.263                10.01
  Sub-total                 |
    D+L pooled SMD          |     -0.363       -0.988        0.263                10.01
---------------------+--------------------------------------------------
        RT1
Hanken et al. (2016) |     -0.629       -1.264        0.007                 9.96
```

Sub-total				
D+L pooled SMD	-0.629	-1.264	0.007	9.96
RT2				
Fiene et al. (2018)	2.295	1.360	3.230	8.41
Sub-total				
D+L pooled SMD	2.295	1.360	3.230	8.41
ANT				
Palm et al. (2016)	0.159	-0.535	0.853	9.66
Chalah et al.（DLPFC	-1.241	-2.206	-0.275	8.24
Chalah et al.(PCC) (	-0.465	-1.354	0.425	8.64
Ayache et al. (2016)	0.205	-0.490	0.900	9.66
Sub-total				
D+L pooled SMD	-0.265	-0.881	0.351	36.21
SDMT				
Grigorescu et al. (2	0.844	-0.075	1.763	8.49
Mattioli et al. (201	0.102	-0.735	0.938	8.92
Salemi et al. (2018)	0.323	-0.636	1.282	8.28
Sub-total				
D+L pooled SMD	0.404	-0.116	0.924	25.69
Overall				
D+L pooled SMD	0.183	-0.320	0.686	100.00

Test(s) of heterogeneity:

	Heterogeneity statistic	degrees of freedom	P	I-squared**	Tau-squared
IVV	0.00	0	.	.%	0.0000
FSAQ	0.00	0	.	.%	0.0000
RT1	0.00	0	.	.%	0.0000
RT2	0.00	0	.	.%	0.0000
ANT	7.12	3	0.068	57.9%	0.2267
SDMT	1.41	2	0.494	0.0%	0.0000
Overall	45.56	10	0.000	78.1%	0.5560

** I-squared: the variation in SMD attributable to heterogeneity)

Note: between group heterogeneity not calculated;
only valid with inverse variance method

Significance test(s) of SMD=0

IVV	z= 2.63	p = 0.008
FSAQ	z= 1.14	p = 0.256
RT1	z= 1.94	p = 0.053
RT2	z= 4.81	p = 0.000
ANT	z= 0.84	p = 0.399
SDMT	z= 1.52	p = 0.127
Overall	z= 0.71	p = 0.475

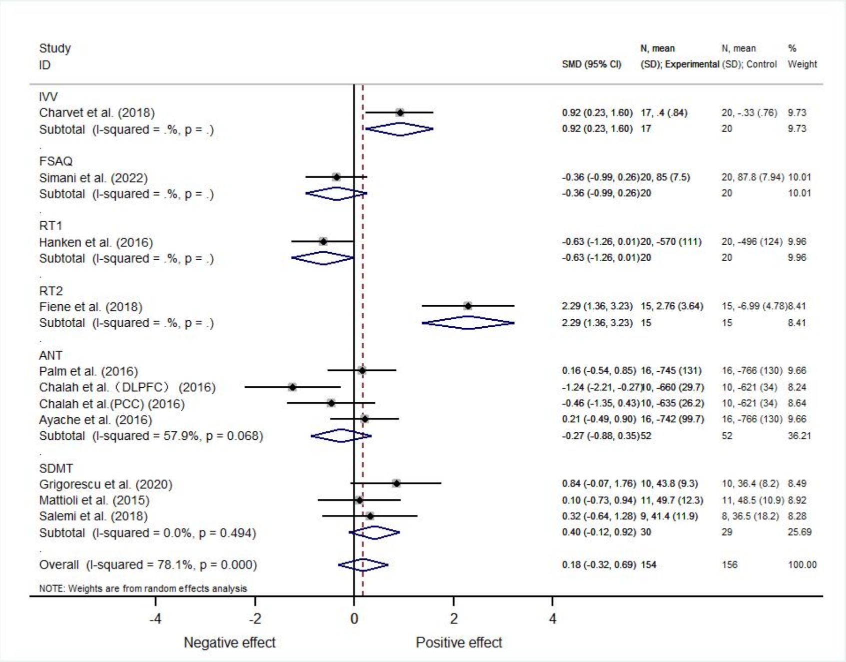

3.1.1.2 Interventions

. metan T_E m_E SD_E T_C m_C SD_C, cohen random label(namevar= Study , yearvar= Year) counts group1(Experimental) group2(Control) texts(200) xlabel(-4,-2,0,2,4) by(Intervention)

Study	SMD	[95% Conf.	Interval]	% Weight
tDCS				
Charvet et al. (2018	0.915	0.234	1.596	9.73
Simani et al. (2022)	-0.363	-0.988	0.263	10.01
Hanken et al. (2016)	-0.629	-1.264	0.007	9.96
Fiene et al. (2018)	2.295	1.360	3.230	8.41
Chalah et al.（DLPFC	-1.241	-2.206	-0.275	8.24
Chalah et al.(PCC) (	-0.465	-1.354	0.425	8.64
Ayache et al. (2016)	0.205	-0.490	0.900	9.66

```
Grigorescu et al. (2 |     0.844      -0.075      1.763              8.49
Mattioli et al. (201 |     0.102      -0.735      0.938              8.92
  Sub-total          |
    D+L pooled SMD   |     0.175      -0.442      0.791              82.06
---------------------+--------------------------------------------------
         tRNS
Palm et al. (2016)   |     0.159      -0.535      0.853              9.66
Salemi et al. (2018) |     0.323      -0.636      1.282              8.28
  Sub-total          |
    D+L pooled SMD   |     0.215      -0.347      0.778              17.94
---------------------+--------------------------------------------------
Overall              |
    D+L pooled SMD   |     0.183      -0.320      0.686              100.00
---------------------+--------------------------------------------------

Test(s) of heterogeneity:
                  Heterogeneity  degrees of
                    statistic     freedom      P     I-squared**   Tau-squared

tDCS                  45.38          8       0.000     82.4%         0.7231
tRNS                   0.07          1       0.786      0.0%         0.0000
Overall               45.56         10       0.000     78.1%         0.5560
** I-squared: the variation in SMD attributable to heterogeneity)

Note: between group heterogeneity not calculated;
only valid with inverse variance method

Significance test(s) of SMD=0

tDCS                   z=   0.56     p = 0.579
tRNS                   z=   0.75     p = 0.453
Overall                z=   0.71     p = 0.475
------------------------------------------------------------------------
```

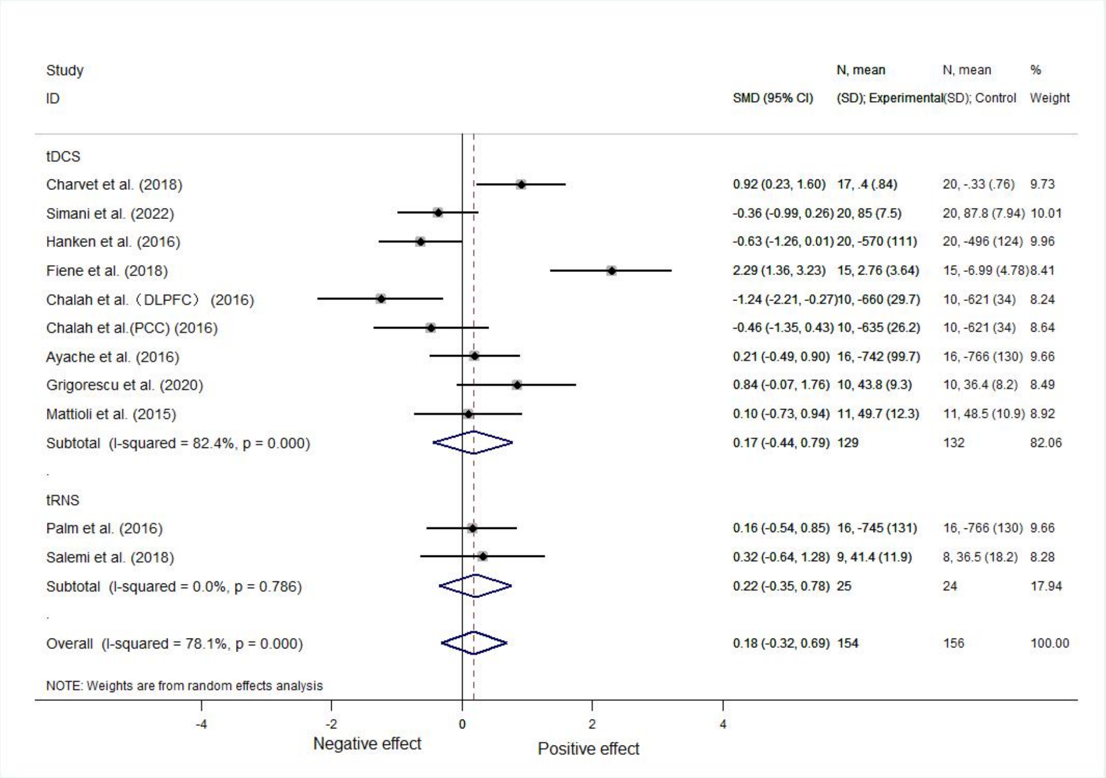

3.1.1.3 Intensity

. metan T_E m_E SD_E T_C m_C SD_C, cohen random label(namevar= Study , yearvar= Year) counts group1(Experimental) group2(Control) texts(200) xlabel(-4,-2,0,2,4) by(Intensity)

Study	SMD	[95% Conf. Interval]		% Weight
1.5mA				
Charvet et al. (2018	0.915	0.234	1.596	9.73
Hanken et al. (2016)	-0.629	-1.264	0.007	9.96
Fiene et al. (2018)	2.295	1.360	3.230	8.41
Salemi et al. (2018)	0.323	-0.636	1.282	8.28
Sub-total				
D+L pooled SMD	0.702	-0.491	1.895	36.37
2mA				
Simani et al. (2022)	-0.363	-0.988	0.263	10.01
Palm et al. (2016)	0.159	-0.535	0.853	9.66
Chalah et al.（DLPFC	-1.241	-2.206	-0.275	8.24
Chalah et al.(PCC) (	-0.465	-1.354	0.425	8.64
Ayache et al. (2016)	0.205	-0.490	0.900	9.66

```
Grigorescu et al. (2 |      0.844      -0.075       1.763              8.49
Mattioli et al. (201 |      0.102      -0.735       0.938              8.92
  Sub-total          |
    D+L pooled SMD   |     -0.090      -0.515       0.335             63.63
---------------------+---------------------------------------------------
Overall              |
    D+L pooled SMD   |      0.183      -0.320       0.686            100.00
---------------------+---------------------------------------------------
```

```
Test(s) of heterogeneity:
                   Heterogeneity  degrees of
                     statistic     freedom      P    I-squared**   Tau-squared

1.5mA                  27.84          3       0.000     89.2%         1.3119
2mA                    12.22          6       0.057     50.9%         0.1648
Overall                45.56         10       0.000     78.1%         0.5560
** I-squared: the variation in SMD attributable to heterogeneity)

Note: between group heterogeneity not calculated;
only valid with inverse variance method

Significance test(s) of SMD=0

1.5mA                     z=   1.15      p = 0.249
2mA                       z=   0.42      p = 0.678
Overall                   z=   0.71      p = 0.475
-----------------------------------------------------------------------
```

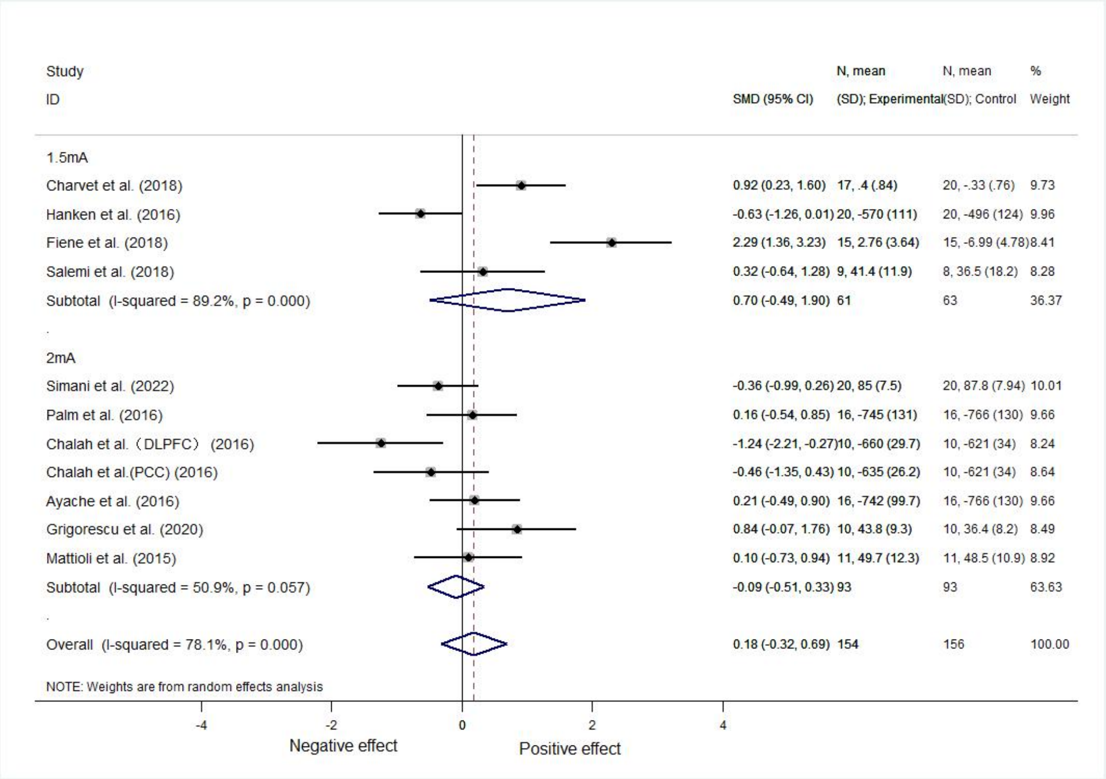

3.1.1.4 Duration

```
. metan T_E m_E SD_E T_C m_C SD_C, cohen random label(namevar= Study , yearvar= Year )
counts group1(Experimental) group2(Control) texts( 180) xlabel(-4,-2,0,2,4) by( Time )

              Study     |     SMD    [95% Conf. Interval]     % Weight
---------------------+---------------------------------------------------
     20min
Charvet et al. (2018 |    0.915      0.234      1.596          10.68
Hanken et al. (2016) |   -0.629     -1.264      0.007          10.90
Chalah et al.（DLPFC |   -1.241     -2.206     -0.275           9.22
Chalah et al.(PCC) ( |   -0.465     -1.354      0.425           9.62
Ayache et al. (2016) |    0.205     -0.490      0.900          10.61
Grigorescu et al. (2 |    0.844     -0.075      1.763           9.47
Mattioli et al. (201 |    0.102     -0.735      0.938           9.90
 Sub-total           |
  D+L pooled SMD     |   -0.021     -0.587      0.545          70.40
---------------------+---------------------------------------------------
     30min
Simani et al. (2022) |   -0.363     -0.988      0.263          10.95
```

Fiene et al. (2018) | 2.295 1.360 3.230 9.39
Sub-total |
D+L pooled SMD | 0.943 -1.661 3.546 20.34
---------------------+--
15min
Salemi et al. (2018) | 0.323 -0.636 1.282 9.26
Sub-total |
D+L pooled SMD | 0.323 -0.636 1.282 9.26
---------------------+--
Overall |
D+L pooled SMD | 0.187 -0.376 0.750 100.00
---------------------+--

Test(s) of heterogeneity:

	Heterogeneity statistic	degrees of freedom	P	I-squared**	Tau-squared
20min	21.71	6	0.001	72.4%	0.4162
30min	21.46	1	0.000	95.3%	3.3667
15min	0.00	0	.	.%	0.0000
Overall	45.55	9	0.000	80.2%	0.6518

** I-squared: the variation in SMD attributable to heterogeneity)

Note: between group heterogeneity not calculated;
only valid with inverse variance method

Significance test(s) of SMD=0

20min	z= 0.07	p = 0.941
30min	z= 0.71	p = 0.478
15min	z= 0.66	p = 0.509
Overall	z= 0.65	p = 0.514

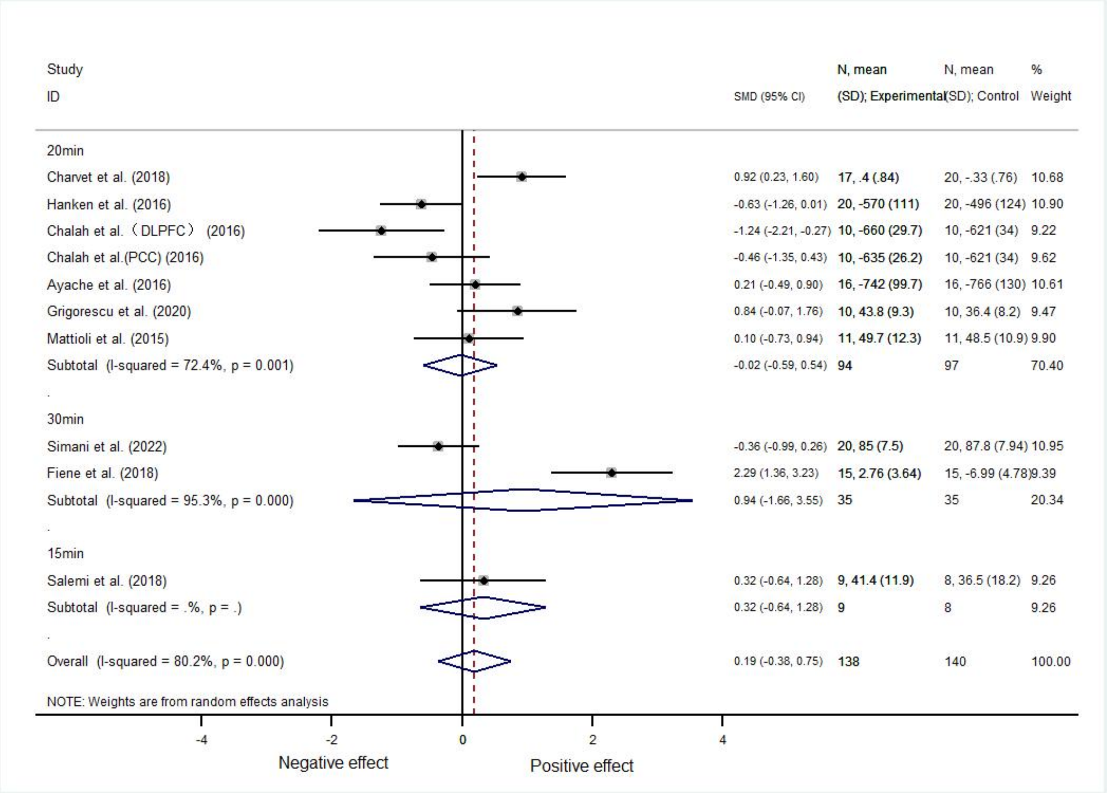

3.1.1.5 Stimulation site

. metan T_E m_E SD_E T_C m_C SD_C, cohen random label(namevar= Study , yearvar= Year) counts group1(Experimental) group2(Control) texts(190) xlabel(-4,-2,0,2,4) by(Location)

```
              Study      |     SMD    [95% Conf. Interval]     % Weight
---------------------+---------------------------------------------------
     Left dorsolateral pr
Charvet et al. (2018 |     0.915      0.234      1.596           9.73
Simani et al. (2022) |    -0.363     -0.988      0.263          10.01
Fiene et al. (2018)  |     2.295      1.360      3.230           8.41
Palm et al. (2016)   |     0.159     -0.535      0.853           9.66
Chalah et al.（DLPFC |    -1.241     -2.206     -0.275           8.24
Ayache et al. (2016) |     0.205     -0.490      0.900           9.66
Grigorescu et al. (2 |     0.844     -0.075      1.763           8.49
Mattioli et al. (201 |     0.102     -0.735      0.938           8.92
Salemi et al. (2018) |     0.323     -0.636      1.282           8.28
  Sub-total          |
   D+L pooled SMD    |     0.352     -0.212      0.915          81.40
---------------------+---------------------------------------------------
     Right parietal corte
```

```
Hanken et al. (2016) |     -0.629       -1.264        0.007                 9.96
Chalah et al.(PCC) ( |     -0.465       -1.354        0.425                 8.64
  Sub-total                |
   D+L pooled SMD        |     -0.573       -1.090       -0.056              18.60
---------------------+--------------------------------------------------
Overall                      |
   D+L pooled SMD        |       0.183       -0.320         0.686              100.00
---------------------+--------------------------------------------------

Test(s) of heterogeneity:
                         Heterogeneity    degrees of
                              statistic         freedom           P        I-squared**      Tau-squared

Left dorsolateral pr 36.52                  8            0.000          78.1%              0.5728
Right parietal corte    0.09                  1            0.769            0.0%              0.0000
Overall                          45.56                 10            0.000          78.1%              0.5560
** I-squared: the variation in SMD attributable to heterogeneity)

Note: between group heterogeneity not calculated;
only valid with inverse variance method

Significance test(s) of SMD=0

Left dorsolateral pr    z=    1.22          p = 0.222
Right parietal corte    z=    2.17          p = 0.030
Overall                          z=    0.71          p = 0.475
-------------------------------------------------------------------------
```

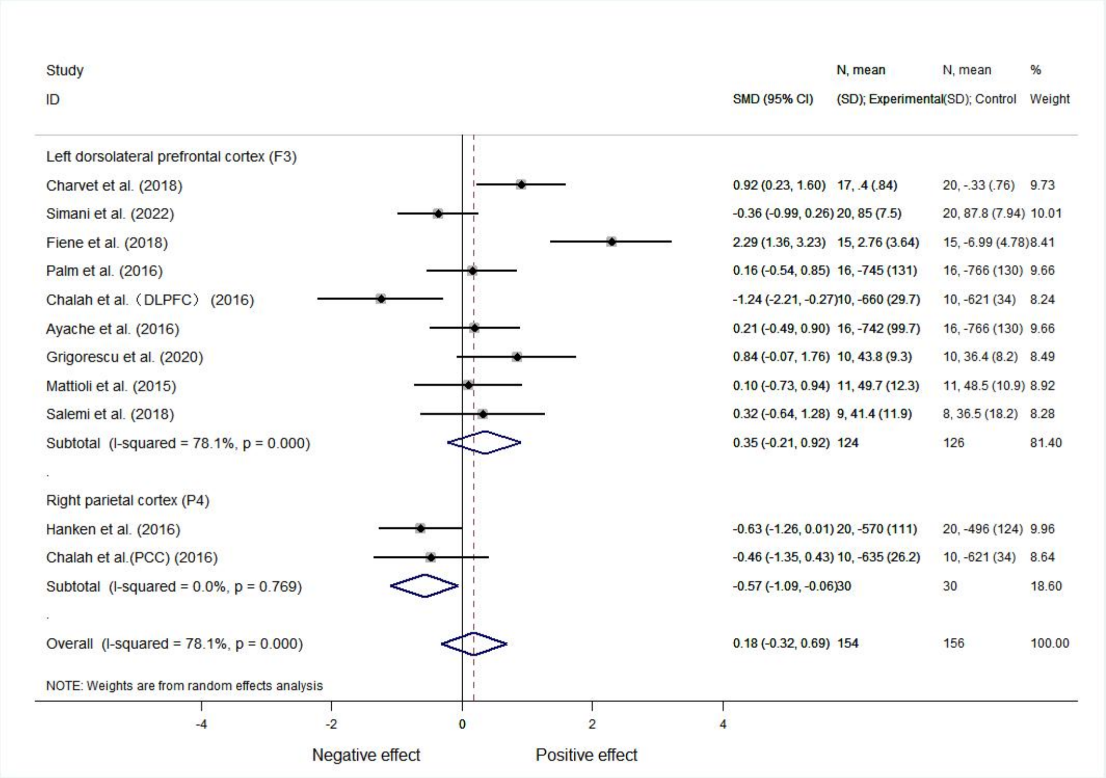

3.1.2 Subgroup Analysis Based on Patient Characteristics

3.1.2.1 Mean age

. metan T_E m_E SD_E T_C m_C SD_C, cohen random label(namevar= Study , yearvar= Year) counts group1(Experimental) group2(Control) texts(180) xlabel(-4,-2,0,2,4) by(Meanage)

Study	SMD	[95% Conf. Interval]		% Weight
age >45				
Charvet et al. (2018	0.915	0.234	1.596	10.62
Hanken et al. (2016)	-0.629	-1.264	0.007	10.86
Palm et al. (2016)	0.159	-0.535	0.853	10.55
Ayache et al. (2016)	0.205	-0.490	0.900	10.55
Sub-total				
D+L pooled SMD	0.155	-0.481	0.791	42.59
age <45				
Simani et al. (2022)	-0.363	-0.988	0.263	10.92
Fiene et al. (2018)	2.295	1.360	3.230	9.24

Chalah et al. (2016) |	-1.241	-2.206	-0.275	9.06
Grigorescu et al. (2 |	0.844	-0.075	1.763	9.32
Mattioli et al. (201 |	0.102	-0.735	0.938	9.78
Salemi et al. (2018) |	0.323	-0.636	1.282	9.10
Sub-total |				
D+L pooled SMD |	0.317	-0.581	1.215	57.41
Overall |				
D+L pooled SMD |	0.245	-0.293	0.783	100.00

Test(s) of heterogeneity:

	Heterogeneity statistic	degrees of freedom	P	I-squared**	Tau-squared
age >45	10.63	3	0.014	71.8%	0.3023
age <45	32.95	5	0.000	84.8%	1.0594
Overall	43.70	9	0.000	79.4%	0.5889

** I-squared: the variation in SMD attributable to heterogeneity)

Note: between group heterogeneity not calculated;
only valid with inverse variance method

Significance test(s) of SMD=0

age >45	z= 0.48	p = 0.633
age <45	z= 0.69	p = 0.489
Overall	z= 0.89	p = 0.372

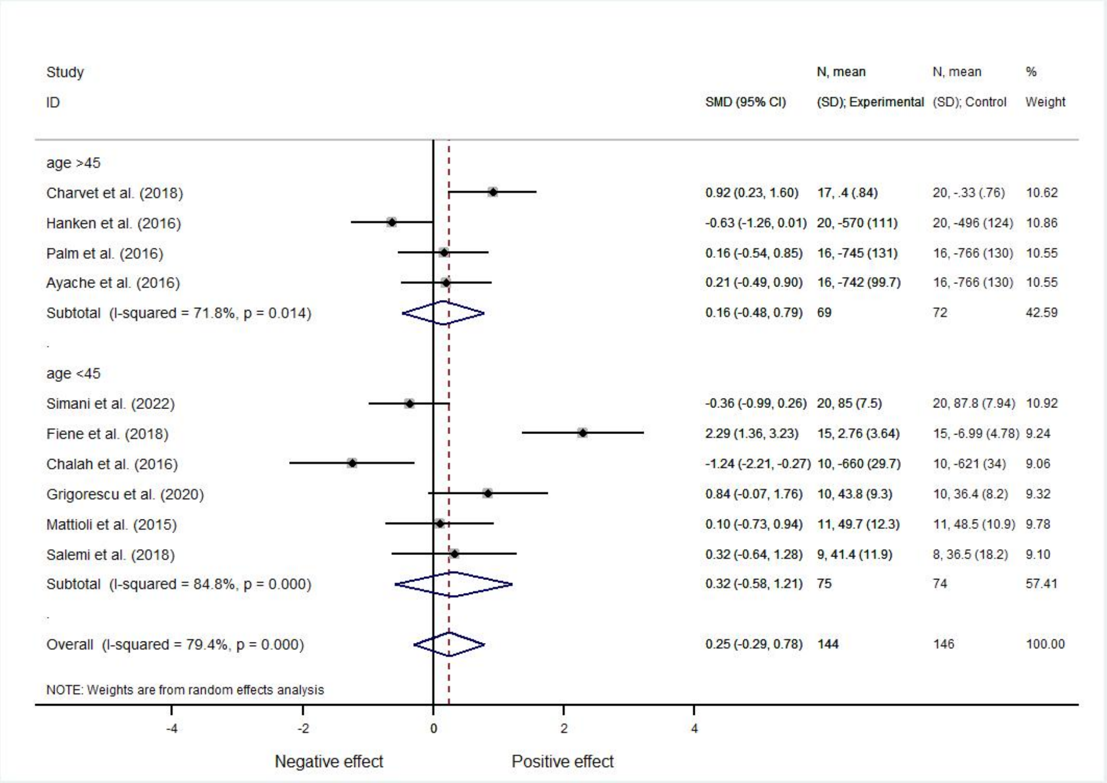

3.1.2.2 Mean EDSS

. metan T_E m_E SD_E T_C m_C SD_C, cohen random label(namevar= Study , yearvar= Year) counts group1(Experimental) group2(Control) texts(200) xlabel(-4,-2,0,2,4) by(MeanEDSS)

Study	SMD	[95% Conf.	Interval]	% Weight
EDSS >3.5				
Hanken et al. (2016)	-0.629	-1.264	0.007	13.66
Fiene et al. (2018)	2.295	1.360	3.230	11.87
Palm et al. (2016)	0.159	-0.535	0.853	13.32
Ayache et al. (2016)	0.205	-0.490	0.900	13.32
Sub-total				
D+L pooled SMD	0.466	-0.601	1.533	52.17
EDSS <3.5				
Chalah et al. (2016)	-1.241	-2.206	-0.275	11.68
Grigorescu et al. (2	0.844	-0.075	1.763	11.97
Mattioli et al. (201	0.102	-0.735	0.938	12.47
Salemi et al. (2018)	0.323	-0.636	1.282	11.72
Sub-total				

D+L pooled SMD | 0.016 -0.825 0.856 47.83

---------------------+--

Overall |

D+L pooled SMD | 0.242 -0.411 0.894 100.00

---------------------+--

Test(s) of heterogeneity:

	Heterogeneity statistic	degrees of freedom	P	I-squared**	Tau-squared
EDSS >3.5	25.81	3	0.000	88.4%	1.0413
EDSS <3.5	10.05	3	0.018	70.2%	0.5152
Overall	36.31	7	0.000	80.7%	0.7066

** I-squared: the variation in SMD attributable to heterogeneity)

Note: between group heterogeneity not calculated;
only valid with inverse variance method

Significance test(s) of SMD=0

EDSS >3.5	z= 0.86	p = 0.392
EDSS <3.5	z= 0.04	p = 0.971
Overall	z= 0.73	p = 0.468

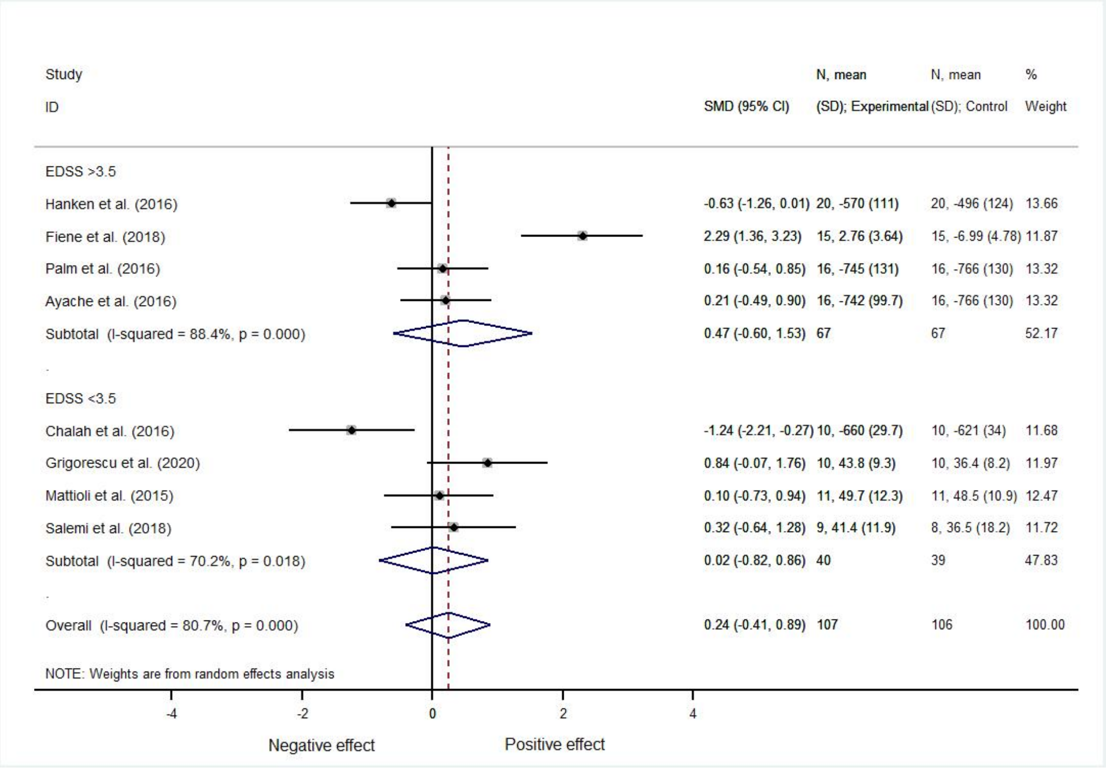

3.1.3 Analysis of the Attention Network Test

```
metan T_E m_E SD_E T_C m_C SD_C, cohen random label(namevar=    Study , yearvar= Year )
counts group1(Experimental) group2(Control) texts(14
> 7) xlabel(-4,-2,0,2,4) by( Outcomes ) nooverall favours(Negative efect#Positive effect)

              Study        |      SMD     [95% Conf. Interval]
---------------------+--------------------------------------------------
        ANT alertness
Ayache et al. (2016) |      0.126       -0.568        0.820
Chalah et al.（DLPFC |       1.368        0.384        2.352
Chalah et al.(PCC) ( |       1.199        0.239        2.159
Palm et al. (2016)   |      0.354       -0.344        1.053
  Sub-total          |
   D+L pooled SMD    |      0.676        0.092        1.259
---------------------+--------------------------------------------------
        ANT orientation
Ayache et al. (2016) |     -0.117       -0.810        0.577
Palm et al. (2016)   |      0.442       -0.260        1.144
Chalah et al.（DLPFC |       0.458       -0.432        1.347
Chalah et al.(PCC) ( |       0.814       -0.102        1.730
  Sub-total          |
   D+L pooled SMD    |      0.336       -0.055        0.726
---------------------+--------------------------------------------------
        ANT mean reaction ti
Ayache et al. (2016) |     -0.454       -1.157        0.248
Chalah et al.（DLPFC |      -1.241       -2.206       -0.275
Chalah et al.(PCC) ( |     -0.465       -1.354        0.425
Palm et al. (2016)   |      0.159       -0.535        0.853
  Sub-total          |
   D+L pooled SMD    |     -0.436       -0.975        0.103
---------------------+--------------------------------------------------
        ANT accuracy
Ayache et al. (2016) |     -0.566       -1.273        0.142
Chalah et al.（DLPFC |       0.000       -0.877        0.877
Chalah et al.(PCC) ( |       0.542       -0.352        1.436
Palm et al. (2016)   |      0.378       -0.322        1.078
  Sub-total          |
   D+L pooled SMD    |      0.063       -0.441        0.567
```

---------------------+--

ANT conflict			
Ayache et al. (2016)		0.159	-0.535	0.853
Chalah et al.（DLPFC		-0.691	-1.596	0.214
Chalah et al.(PCC) (		-0.506	-1.398	0.386
Palm et al. (2016)		0.023	-0.670	0.716
Sub-total				
D+L pooled SMD		-0.166	-0.554	0.222

---------------------+--

Test(s) of heterogeneity:

	Heterogeneity statistic	degrees of freedom	P	I-squared**	Tau-squared
ANT alertness	6.10	3	0.107	50.8%	0.1786
ANT orientation	2.84	3	0.417	0.0%	0.0000
ANT mean reaction ti	5.44	3	0.142	44.9%	0.1351
ANT accuracy	4.93	3	0.177	39.1%	0.1032
ANT conflict	2.98	3	0.394	0.0%	0.0000

** I-squared: the variation in SMD attributable to heterogeneity)

Significance test(s) of SMD=0

ANT alertness	z= 2.27	p = 0.023
ANT orientation	z= 1.69	p = 0.092
ANT mean reaction ti	z= 1.58	p = 0.113
ANT accuracy	z= 0.25	p = 0.806
ANT conflict	z= 0.84	p = 0.402

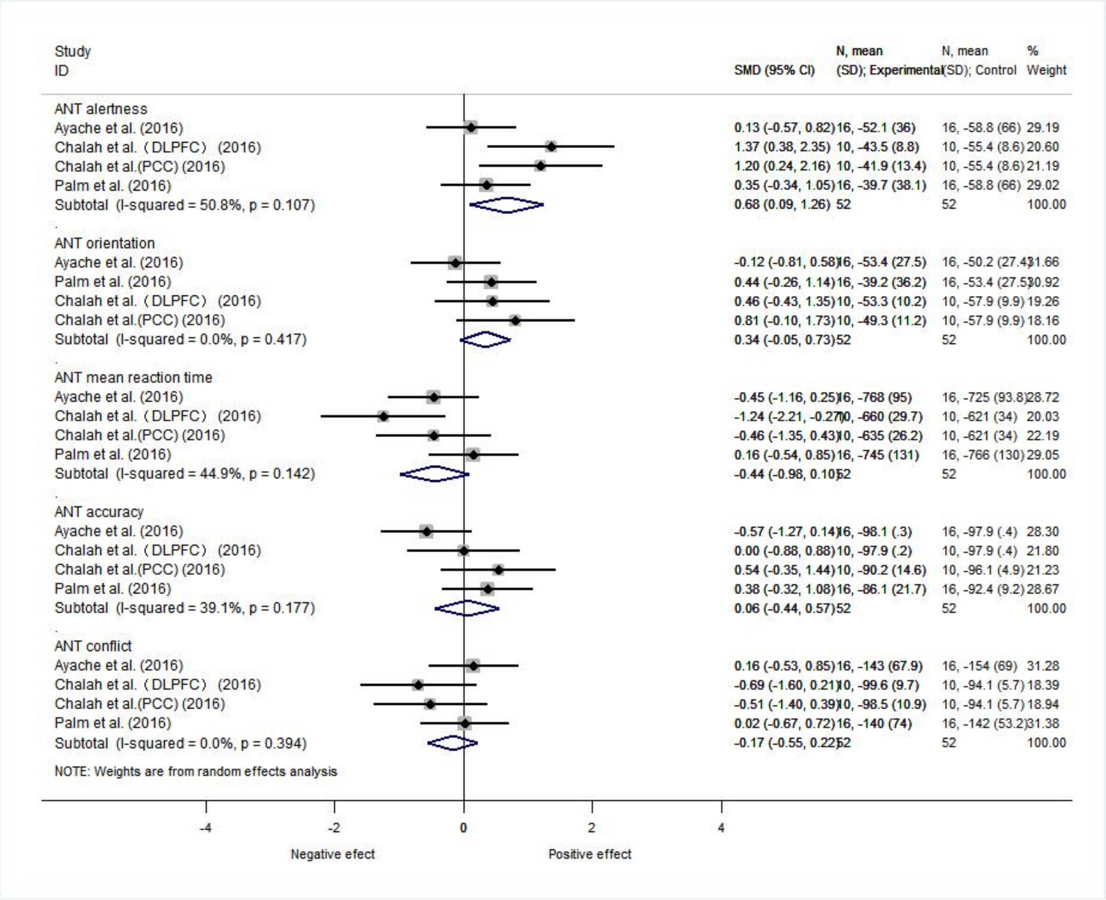

3.2 Motor function

. metan T_E m_E SD_E T_C m_C SD_C, cohen fixed label(namevar= Study , yearvar= Year) counts group1(Experimental) group2(Control) texts(230) xlabel(-1,0,1,2)

Study	SMD	[95% Conf. Interval]		% Weight
Baroni et al. (2022)	-0.058	-1.038	0.922	11.37
San et al. (2019)	1.281	0.163	2.399	8.74
Salemi et al. (2019)	0.583	-0.392	1.558	11.49
Pilloni et al.A (202	0.058	-0.895	1.010	12.04
Pilloni et al.B (202	0.205	-0.831	1.241	10.18
Darwish et al. (2019	0.965	0.206	1.724	18.96
Iodice et al. (2015)	0.214	-0.666	1.093	14.13
Mori et al. (2011)	0.791	-0.123	1.704	13.08
I-V pooled SMD	0.517	0.186	0.847	100.00

--------------------+--

Heterogeneity chi-squared = 6.52 (d.f. = 7) p = 0.481
I-squared (variation in SMD attributable to heterogeneity) = 0.0%

Test of SMD=0 : z= 3.07 p = 0.002

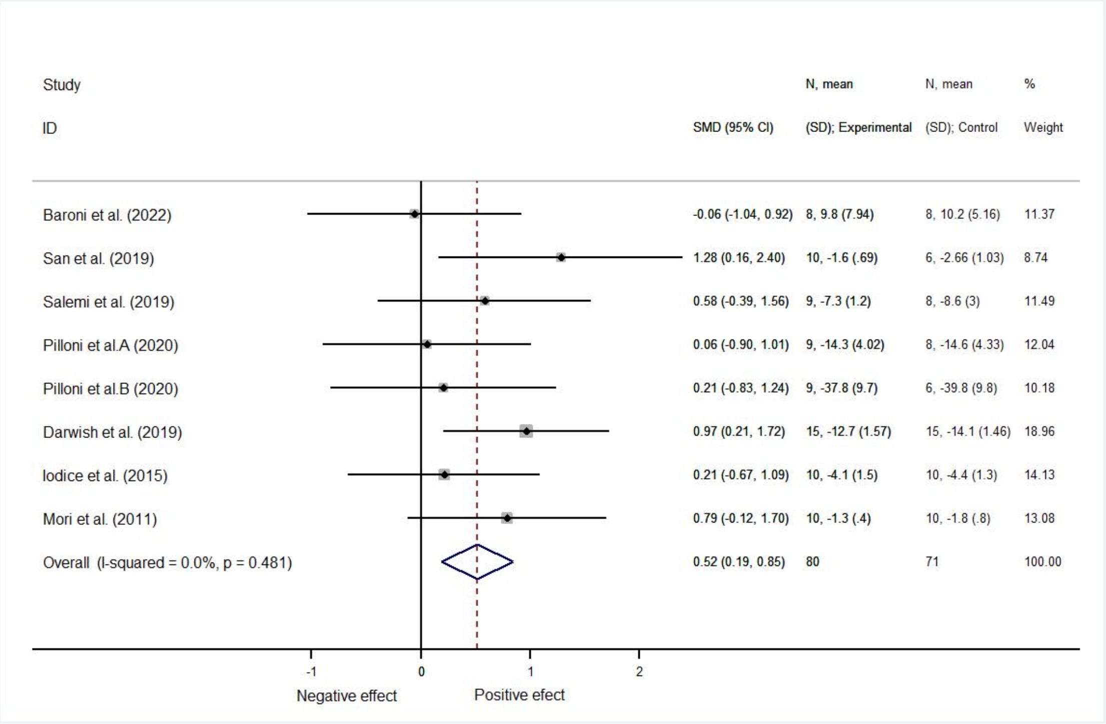

3.2.1 Subgroup Analysis Based on Treatment Modality

3.2.1.1 Outcome indicators

. metan T_E m_E SD_E T_C m_C SD_C, cohen fixed label(namevar= Study , yearvar= Year) counts group1(Experimental) group2(Control) texts(150) xlabel(-1,0,1,2) by(Outcomes)

Study	SMD	[95% Conf. Interval]		% Weight
MSWS12				
Baroni et al. (2022)	-0.058	-1.038	0.922	11.37
Pilloni et al.B (202	0.205	-0.831	1.241	10.18
Sub-total				
I-V pooled SMD	0.066	-0.646	0.778	21.55
MAS/PSFS				

San et al. (2019)	1.281	0.163	2.399	8.74
Iodice et al. (2015)	0.214	-0.666	1.093	14.13
Mori et al. (2011)	0.791	-0.123	1.704	13.08
Sub-total				
I-V pooled SMD	0.683	0.132	1.234	35.96
T25FWT				
Salemi et al. (2019)	0.583	-0.392	1.558	11.49
Sub-total				
I-V pooled SMD	0.583	-0.392	1.558	11.49
TUG time				
Pilloni et al.A (202	0.058	-0.895	1.010	12.04
Sub-total				
I-V pooled SMD	0.058	-0.895	1.010	12.04
5STS				
Darwish et al. (2019	0.965	0.206	1.724	18.96
Sub-total				
I-V pooled SMD	0.965	0.206	1.724	18.96
Overall				
I-V pooled SMD	0.517	0.186	0.847	100.00

Test(s) of heterogeneity:

	Heterogeneity statistic	degrees of freedom	P	I-squared**
MSWS12	0.13	1	0.717	0.0%
MAS/PSFS	2.25	2	0.325	11.0%
T25FWT	0.00	0	.	.%
TUG time	0.00	0	.	.%
5STS	0.00	0	.	.%
Overall	6.52	7	0.481	0.0%
Overall Test for heterogeneity between sub-groups:				
	4.14	4	0.387	

** I-squared: the variation in SMD attributable to heterogeneity)

Some heterogeneity observed (up to 11.0%) in one or more sub-groups,
Test for heterogeneity between sub-groups may be invalid

Significance test(s) of SMD=0

MSWS12 z= 0.18 p = 0.855
MAS/PSFS z= 2.43 p = 0.015
T25FWT z= 1.17 p = 0.241
TUG time z= 0.12 p = 0.906
5STS z= 2.49 p = 0.013
Overall z= 3.07 p = 0.002

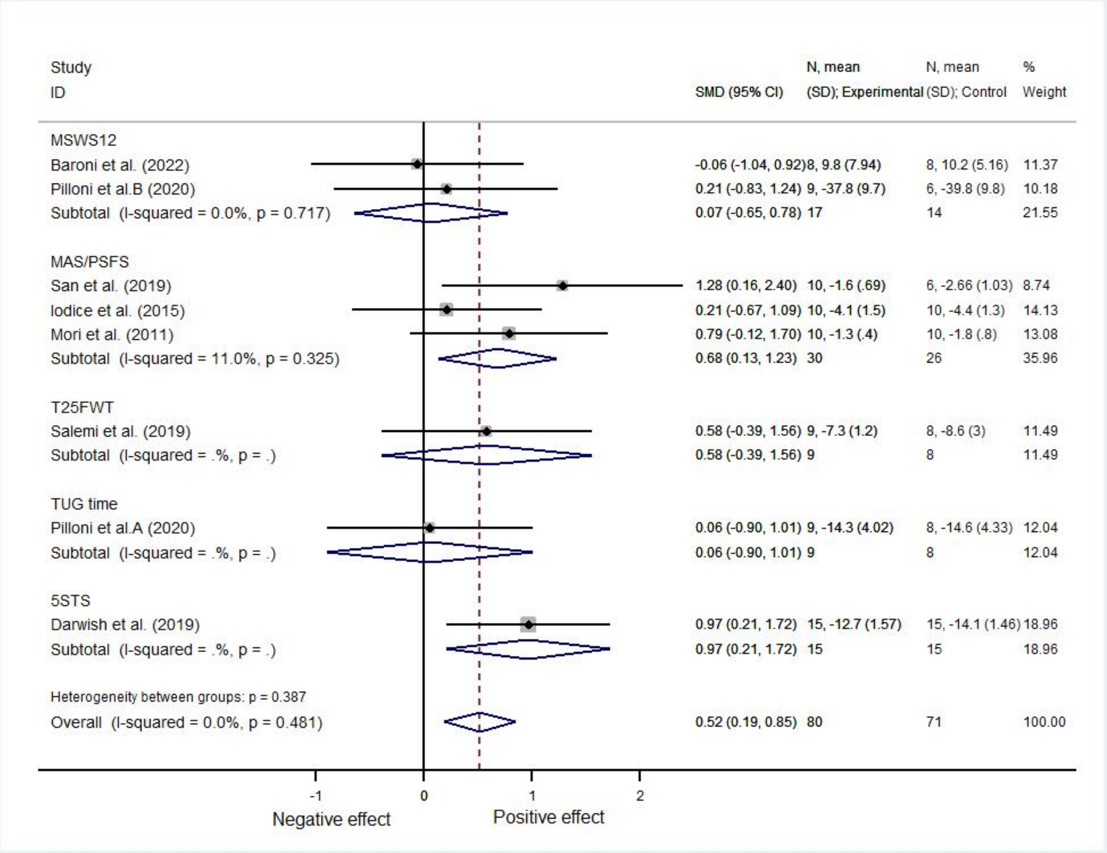

3.2.1.2 Interventions

. metan T_E m_E SD_E T_C m_C SD_C, cohen fixed label(namevar= Study , yearvar= Year) counts group1(Experimental) group2(Control) texts(190) xlabel(-1,0,1,2) by(Intervention)

Study	SMD	[95% Conf.	Interval]	% Weight
TES				
Baroni et al. (2022)	-0.058	-1.038	0.922	11.37
Salemi et al. (2019)	0.583	-0.392	1.558	11.49
Pilloni et al.A (202	0.058	-0.895	1.010	12.04
Pilloni et al.B (202	0.205	-0.831	1.241	10.18
Iodice et al. (2015)	0.214	-0.666	1.093	14.13

```
 Sub-total              |
   I-V pooled SMD       |     0.200     -0.229      0.630          59.21
---------------------+---------------------------------------------------
        TMS
San et al. (2019)    |     1.281      0.163      2.399           8.74
Darwish et al. (2019 |     0.965      0.206      1.724          18.96
Mori et al. (2011)   |     0.791     -0.123      1.704          13.08
 Sub-total              |
   I-V pooled SMD       |     0.977      0.459      1.494          40.79
---------------------+---------------------------------------------------
Overall                 |
   I-V pooled SMD       |     0.517      0.186      0.847         100.00
---------------------+---------------------------------------------------

Test(s) of heterogeneity:
                   Heterogeneity  degrees of
                     statistic     freedom      P     I-squared**
TES                     0.95          4       0.918      0.0%
TMS                     0.44          2       0.801      0.0%
Overall                 6.52          7       0.481      0.0%
Overall Test for heterogeneity between sub-groups:
                        5.13          1       0.024

** I-squared: the variation in SMD attributable to heterogeneity)


Significance test(s) of SMD=0

TES                  z=   0.91     p = 0.361
TMS                  z=   3.70     p = 0.000
Overall              z=   3.07     p = 0.002
-----------------------------------------------------------------------
```

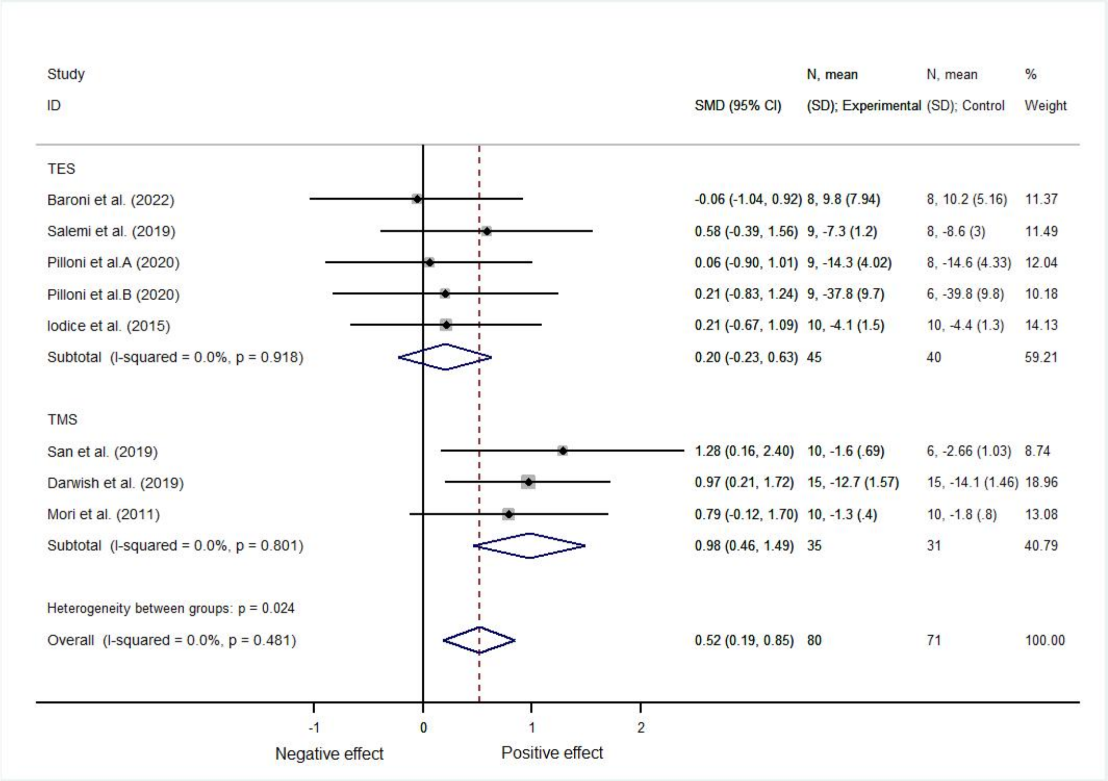

3.2.1.3 Intensity

. metan T_E m_E SD_E T_C m_C SD_C, cohen random label(namevar= Study , yearvar= Year) counts group1(Experimental) group2(Control) texts(180) xlabel(-4,-2,0,2,4) by (Intensity)

```
           Study     |     SMD   [95% Conf. Interval]     % Weight
---------------------+---------------------------------------------------
    2mA
Baroni et al. (2022) |  -0.058    -1.038     0.922         19.20
Iodice et al. (2015) |   0.214    -0.666     1.093         23.86
  Sub-total          |
   D+L pooled SMD    |   0.092    -0.562     0.747         43.07
---------------------+---------------------------------------------------
    1.5mA
Salemi et al. (2019) |   0.583    -0.392     1.558         19.41
  Sub-total          |
   D+L pooled SMD    |   0.583    -0.392     1.558         19.41
---------------------+---------------------------------------------------
    2.5mA
Pilloni et al.A (202 |   0.058    -0.895     1.010         20.33
```

Pilloni et al.B (202	0.205	-0.831	1.241	17.19
Sub-total				
D+L pooled SMD	0.125	-0.576	0.827	37.52
Overall				
D+L pooled SMD	0.200	-0.229	0.630	100.00

Test(s) of heterogeneity:

	Heterogeneity statistic	degrees of freedom	P	I-squared**	Tau-squared
2mA	0.16	1	0.686	0.0%	0.0000
1.5mA	0.00	0	.	.%	0.0000
2.5mA	0.04	1	0.837	0.0%	0.0000
Overall	0.95	4	0.918	0.0%	0.0000

** I-squared: the variation in SMD attributable to heterogeneity)

Note: between group heterogeneity not calculated;
only valid with inverse variance method

Significance test(s) of SMD=0

2mA	z= 0.28	p = 0.782
1.5mA	z= 1.17	p = 0.241
2.5mA	z= 0.35	p = 0.726
Overall	z= 0.91	p = 0.361

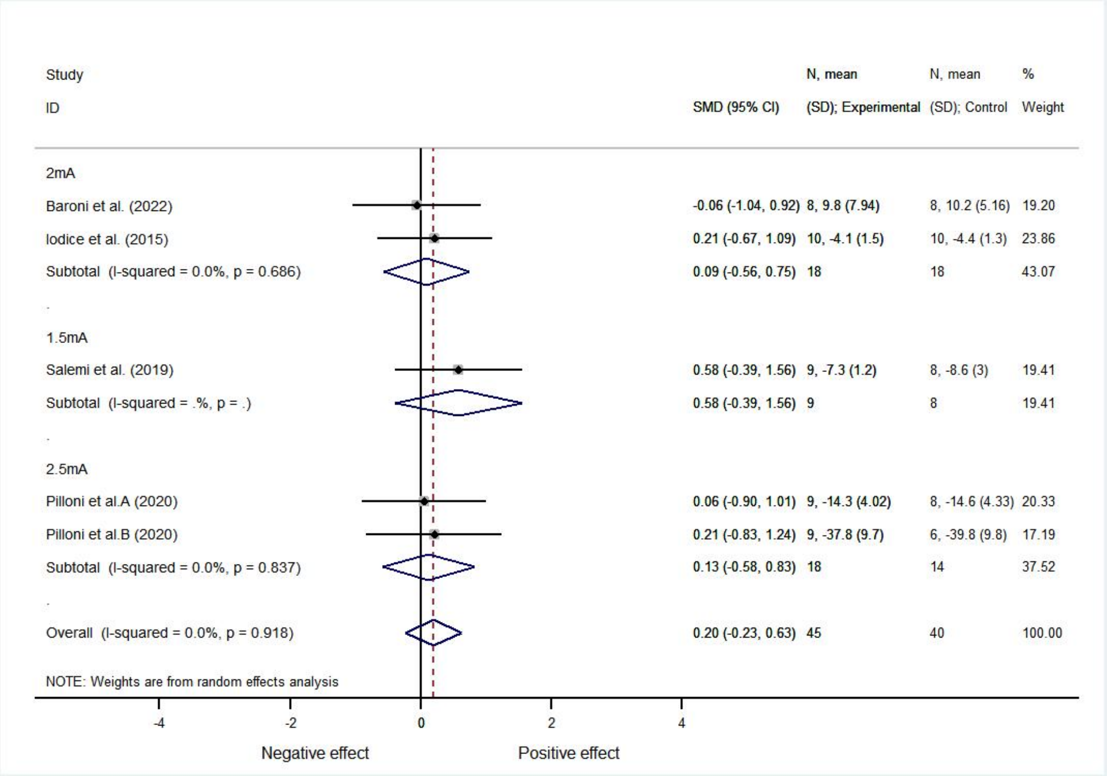

3.2.1.4 Duration

. metan T_E m_E SD_E T_C m_C SD_C, cohen fixed label(namevar= Study , yearvar= Year) counts group1(Experimental) group2(Control) texts(200) xlabel(-1,0,1,2) by(Time)

Study	SMD	[95% Conf. Interval]		% Weight
15min				
Baroni et al. (2022)	-0.058	-1.038	0.922	16.73
San et al. (2019)	1.281	0.163	2.399	12.87
Salemi et al. (2019)	0.583	-0.392	1.558	16.91
Sub-total				
I-V pooled SMD	0.545	-0.042	1.133	46.51
20min				
Pilloni et al.A (202	0.058	-0.895	1.010	17.72
Pilloni et al.B (202	0.205	-0.831	1.241	14.98
Iodice et al. (2015)	0.214	-0.666	1.093	20.79
Sub-total				
I-V pooled SMD	0.160	-0.389	0.708	53.49

```
Overall                     |
   I-V pooled SMD           |     0.339      -0.062       0.740            100.00
---------------------+---------------------------------------------------
```

Test(s) of heterogeneity:

	Heterogeneity statistic	degrees of freedom	P	I-squared**
15min	3.13	2	0.210	36.0%
20min	0.07	2	0.967	0.0%
Overall	4.08	5	0.539	0.0%
Overall Test for heterogeneity between sub-groups:				
	0.88	1	0.347	

** I-squared: the variation in SMD attributable to heterogeneity)

Some heterogeneity observed (up to 36.0%) in one or more sub-groups,
Test for heterogeneity between sub-groups may be invalid

Significance test(s) of SMD=0

15min	z= 1.82	p = 0.069
20min	z= 0.57	p = 0.568
Overall	z= 1.66	p = 0.097

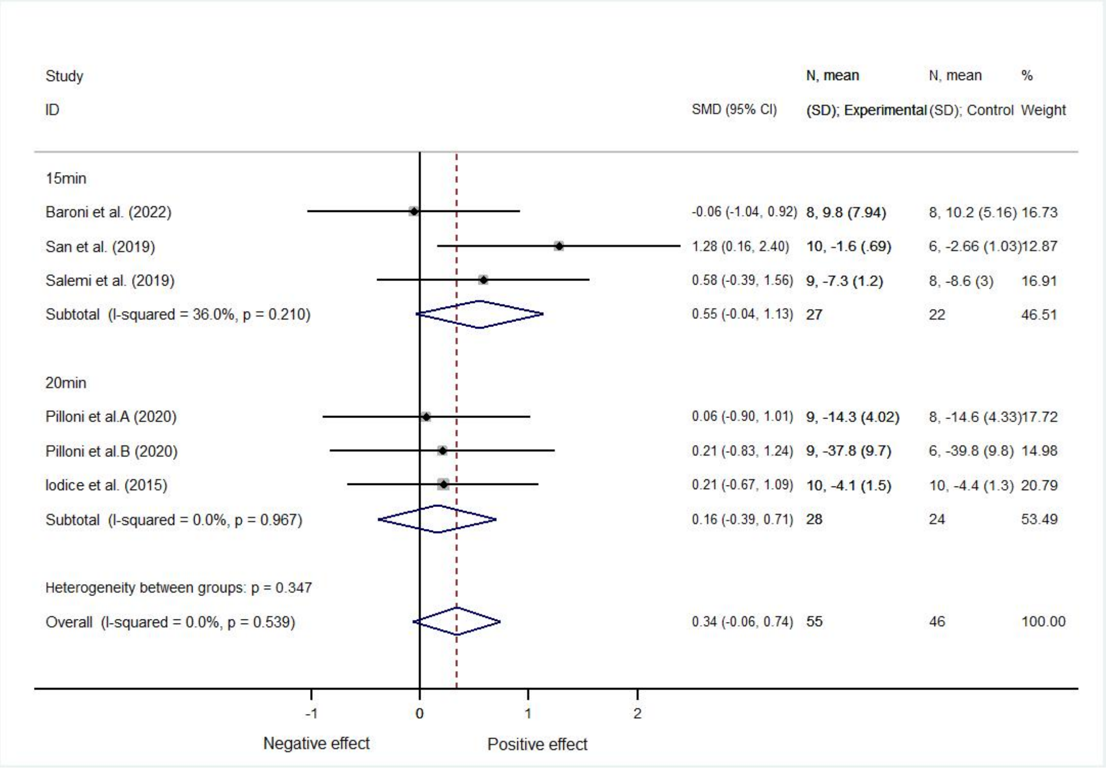

3.2.1.5 Stimulation site

```
. metan T_E m_E SD_E T_C m_C SD_C, cohen random label(namevar=    Study , yearvar= Year )
counts group1(Experimental) group2(Control) texts(165) xlabel(-1,0,1,2) by( location )

            Study     |     SMD   [95% Conf. Interval]     % Weight
---------------------+--------------------------------------------------
     right cerebellar cor
Baroni et al. (2022) |   -0.058    -1.038     0.922          11.37
  Sub-total          |
    D+L pooled SMD   |   -0.058    -1.038     0.922          11.37
---------------------+--------------------------------------------------
     lower extremity moto
San et al. (2019)    |    1.281     0.163     2.399           8.74
  Sub-total          |
    D+L pooled SMD   |    1.281     0.163     2.399           8.74
---------------------+--------------------------------------------------
     motor cortex M1
Salemi et al. (2019) |    0.583    -0.392     1.558          11.49
Pilloni et al.A (202 |    0.058    -0.895     1.010          12.04
Pilloni et al.B (202 |    0.205    -0.831     1.241          10.18
Darwish et al. (2019 |    0.965     0.206     1.724          18.96
Iodice et al. (2015) |    0.214    -0.666     1.093          14.13
Mori et al. (2011)   |    0.791    -0.123     1.704          13.08
  Sub-total          |
    D+L pooled SMD   |    0.515     0.145     0.885          79.89
---------------------+--------------------------------------------------
Overall              |
    D+L pooled SMD   |    0.517     0.186     0.847         100.00
---------------------+--------------------------------------------------

Test(s) of heterogeneity:
                      Heterogeneity  degrees of
                        statistic     freedom      P    I-squared**   Tau-squared

right cerebellar cor     0.00           0          .        .%         0.0000
lower extremity moto     0.00           0          .        .%         0.0000
motor cortex M1          3.40           5        0.639     0.0%        0.0000
Overall                  6.52           7        0.481     0.0%        0.0000
** I-squared: the variation in SMD attributable to heterogeneity)

Note: between group heterogeneity not calculated;
```

only valid with inverse variance method

Significance test(s) of SMD=0

```
right cerebellar cor    z=    0.12         p = 0.907
lower extremity moto    z=    2.25         p = 0.025
motor cortex M1         z=    2.73         p = 0.006
Overall                 z=    3.07         p = 0.002
------------------------------------------------------------------------
```

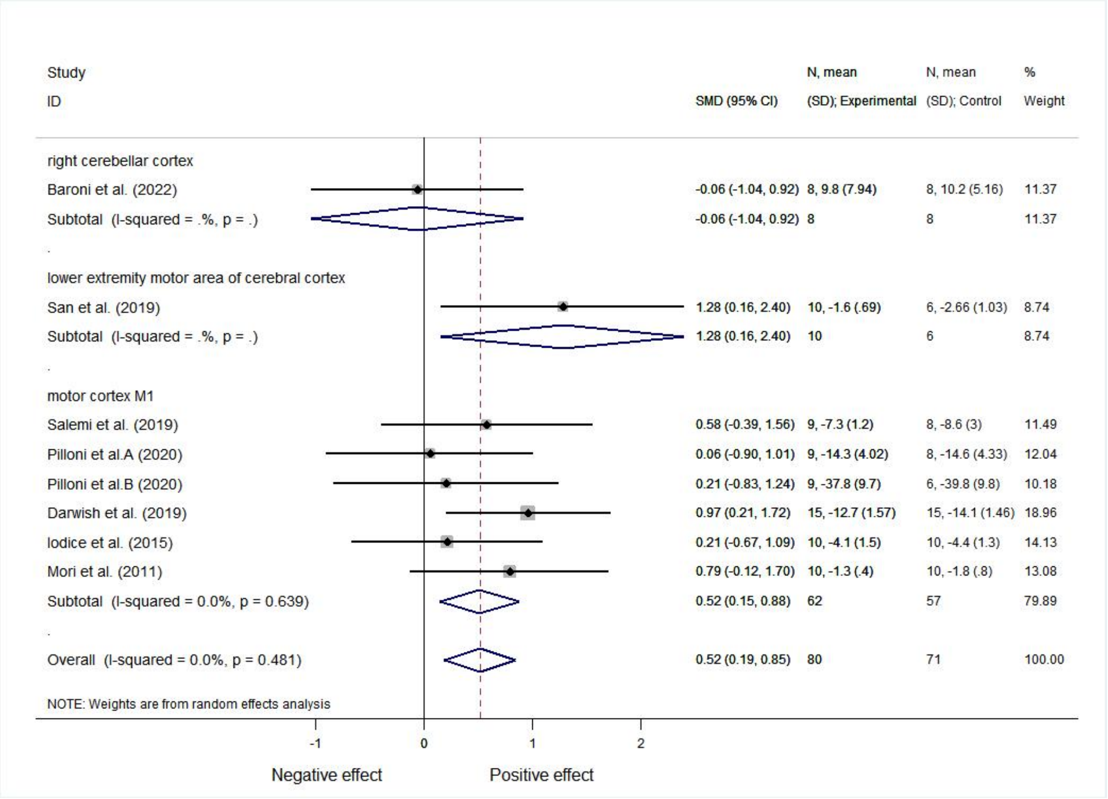

3.2.2 Subgroup Analysis Based on Patient Characteristics

3.2.2.1 Mean age

. metan T_E m_E SD_E T_C m_C SD_C, cohen fixed label(namevar= Study , yearvar= Year) counts group1(Experimental) group2(Control) texts(180) xlabel(-4,-2,0,2,4) by(Meanage)

```
              Study       |        SMD     [95% Conf. Interval]        % Weight
---------------------+---------------------------------------------------
```

age >45				
Baroni et al. (2022)	-0.058	-1.038	0.922	11.37
San et al. (2019)	1.281	0.163	2.399	8.74
Pilloni et al.A (202	0.058	-0.895	1.010	12.04
Pilloni et al.B (202	0.205	-0.831	1.241	10.18
Sub-total				
I-V pooled SMD	0.315	-0.193	0.823	42.33
age <45				
Salemi et al. (2019)	0.583	-0.392	1.558	11.49
Darwish et al. (2019	0.965	0.206	1.724	18.96
Iodice et al. (2015)	0.214	-0.666	1.093	14.13
Mori et al. (2011)	0.791	-0.123	1.704	13.08
Sub-total				
I-V pooled SMD	0.665	0.230	1.101	57.67
Overall				
I-V pooled SMD	0.517	0.186	0.847	100.00

Test(s) of heterogeneity:

	Heterogeneity statistic	degrees of freedom	P	I-squared**
age >45	3.75	3	0.290	20.0%
age <45	1.71	3	0.634	0.0%
Overall	6.52	7	0.481	0.0%
Overall Test for heterogeneity between sub-groups:	1.06	1	0.304	

** I-squared: the variation in SMD attributable to heterogeneity)

Some heterogeneity observed (up to 20.0%) in one or more sub-groups,
Test for heterogeneity between sub-groups may be invalid

Significance test(s) of SMD=0

age >45	z= 1.21	p = 0.225
age <45	z= 3.00	p = 0.003
Overall	z= 3.07	p = 0.002

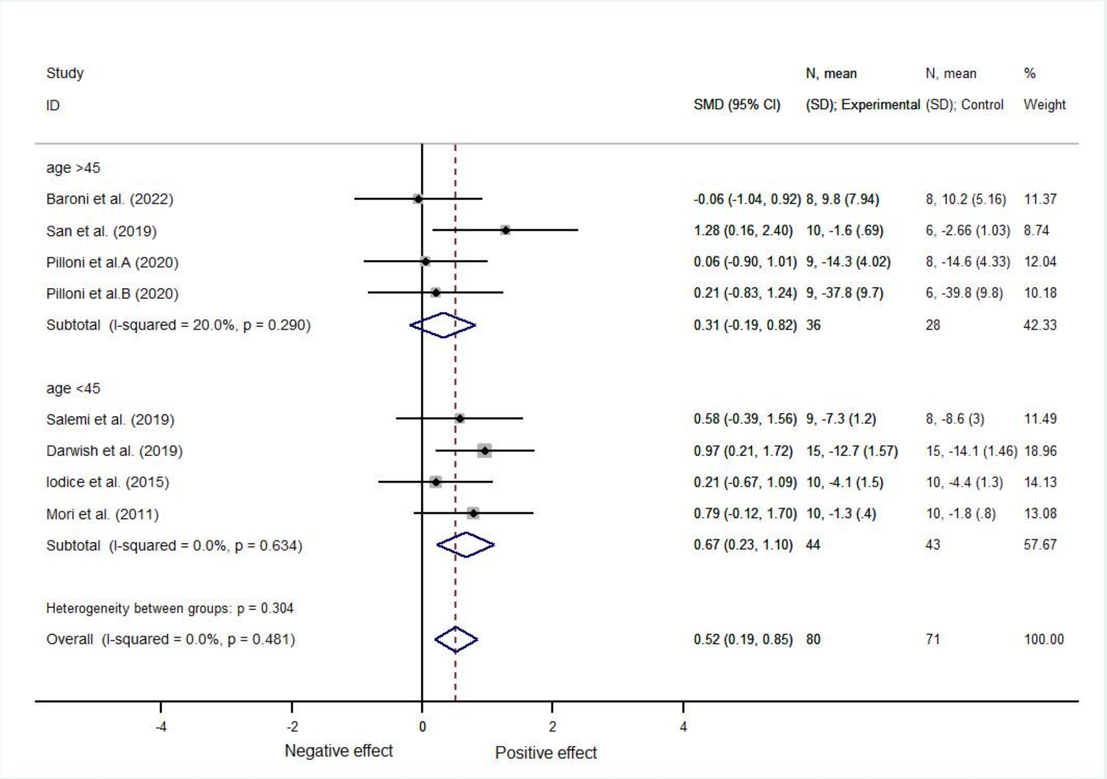

3.2.2.2 Mean EDSS

. metan T_E m_E SD_E T_C m_C SD_C, cohen fixed label(namevar= Study , yearvar= Year) counts group1(Experimental) group2(Control) texts(180) xlabel(-4,-2,0,2,4) by(MeanEDSS)

Study	SMD	[95% Conf. Interval]		% Weight
EDSS >3.5				
Baroni et al. (2022)	-0.058	-1.038	0.922	12.46
Pilloni et al.A (202	0.058	-0.895	1.010	13.19
Pilloni et al.B (202	0.205	-0.831	1.241	11.15
Iodice et al. (2015)	0.214	-0.666	1.093	15.48
Mori et al. (2011)	0.791	-0.123	1.704	14.34
Sub-total				
I-V pooled SMD	0.255	-0.169	0.679	66.63
EDSS <3.5				
Salemi et al. (2019)	0.583	-0.392	1.558	12.59
Darwish et al. (2019	0.965	0.206	1.724	20.78
Sub-total				
I-V pooled SMD	0.821	0.222	1.420	33.37

---------------------+--

Overall				
I-V pooled SMD	0.444	0.098	0.790	100.00

---------------------+--

Test(s) of heterogeneity:

	Heterogeneity statistic	degrees of freedom	P	I-squared**
EDSS >3.5	1.89	4	0.755	0.0%
EDSS <3.5	0.37	1	0.544	0.0%
Overall	4.55	6	0.603	0.0%
Overall Test for heterogeneity between sub-groups:				
	2.29	1	0.130	

** I-squared: the variation in SMD attributable to heterogeneity)

Significance test(s) of SMD=0

EDSS >3.5	z= 1.18	p = 0.239
EDSS <3.5	z= 2.69	p = 0.007
Overall	z= 2.51	p = 0.012

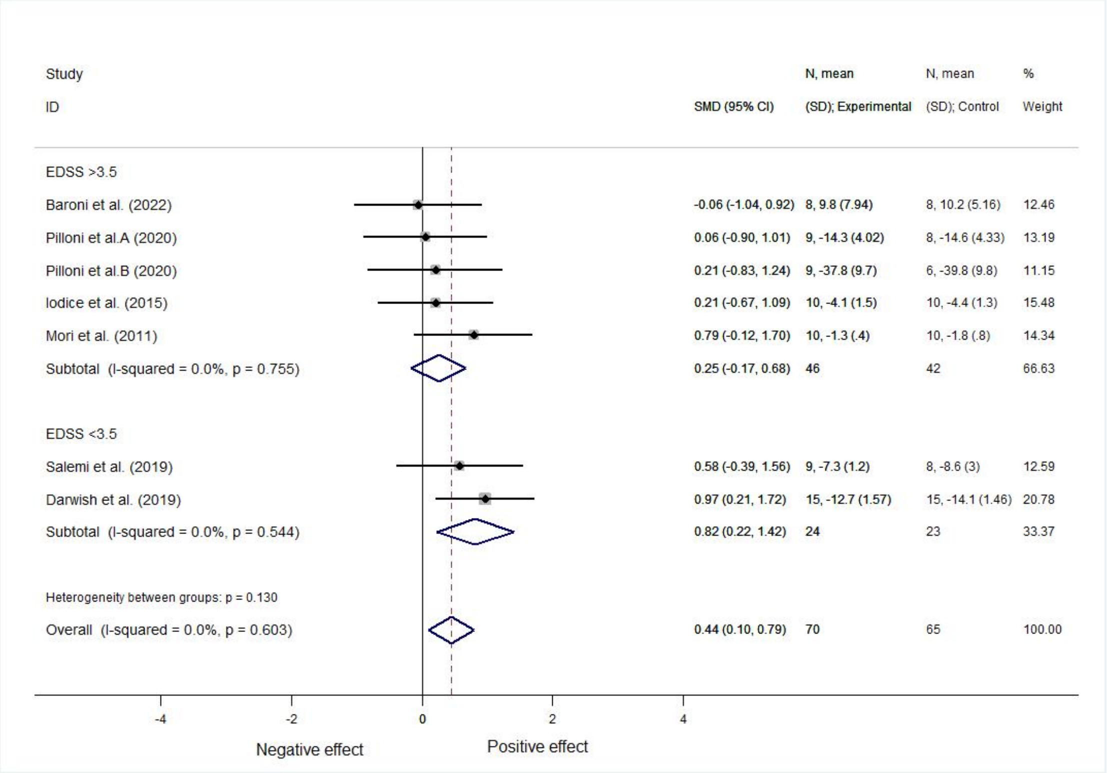

3.3 Meta regression

3.3.1.1 Mean Age of Cognition

. metareg _ES Age , wsse(_seES) eform graph

Meta-regression Number of obs = 10
REML estimate of between-study variance tau2 = .7543
% residual variation due to heterogeneity I-squared_res = 80.42%
Proportion of between-study variance explained Adj R-squared = -9.67%
With Knapp-Hartung modification

_ES	exp(b)	Std. Err.	t	P>|t|	[95% Conf. Interval]	
Age	1.039127	.0600732	0.66	0.525	.9094344	1.187314
_cons	.234013	.6037808	-0.56	0.589	.0006099	89.78172

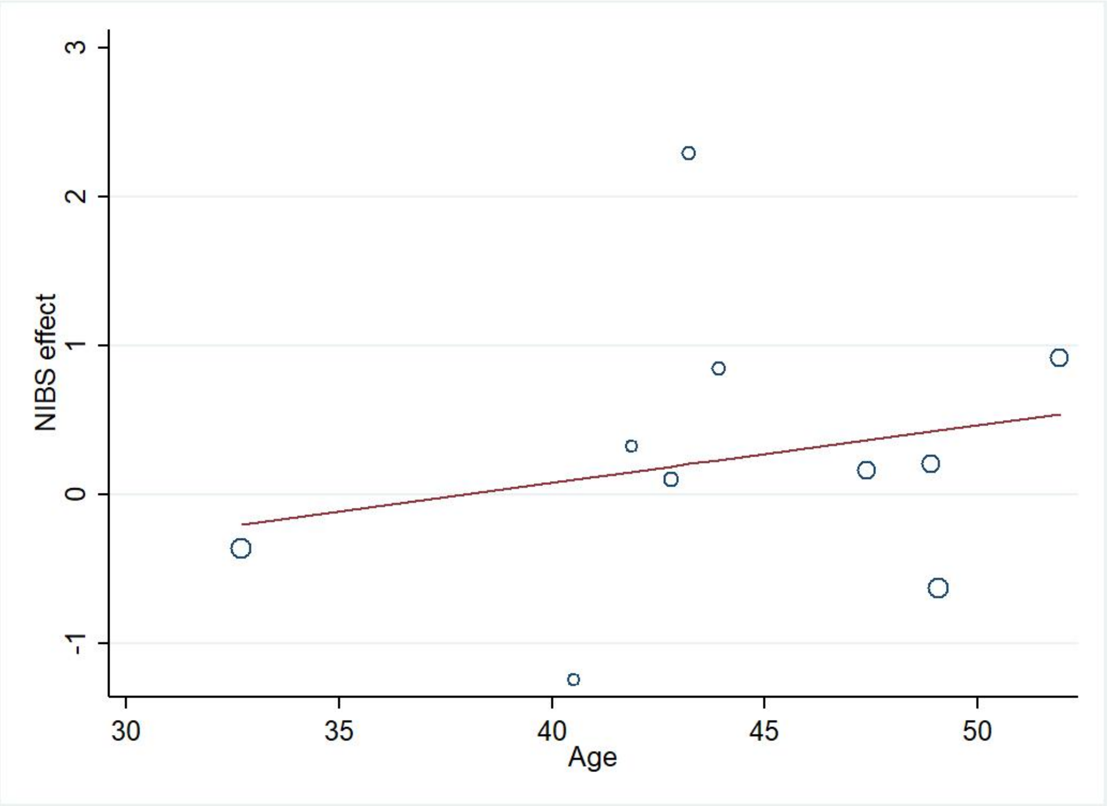

3.3.1.2 Mean EDSS of Cognition

```
. metareg _ES EDSS , wsse( _seES ) eform graph

Meta-regression                                          Number of obs   =          8
REML estimate of between-study variance                   tau2            =      .9901
% residual variation due to heterogeneity                 I-squared_res   =     83.46%
Proportion of between-study variance explained            Adj R-squared   =   -19.60%
With Knapp-Hartung modification
------------------------------------------------------------------------------
         _ES |     exp(b)   Std. Err.      t    P>|t|     [95% Conf. Interval]
-------------+----------------------------------------------------------------
        EDSS |   1.195567   .5823211     0.37   0.726      .3630592    3.937044
       _cons |   .7036166    1.17767    -0.21   0.841      .0117135    42.26535
------------------------------------------------------------------------------
```

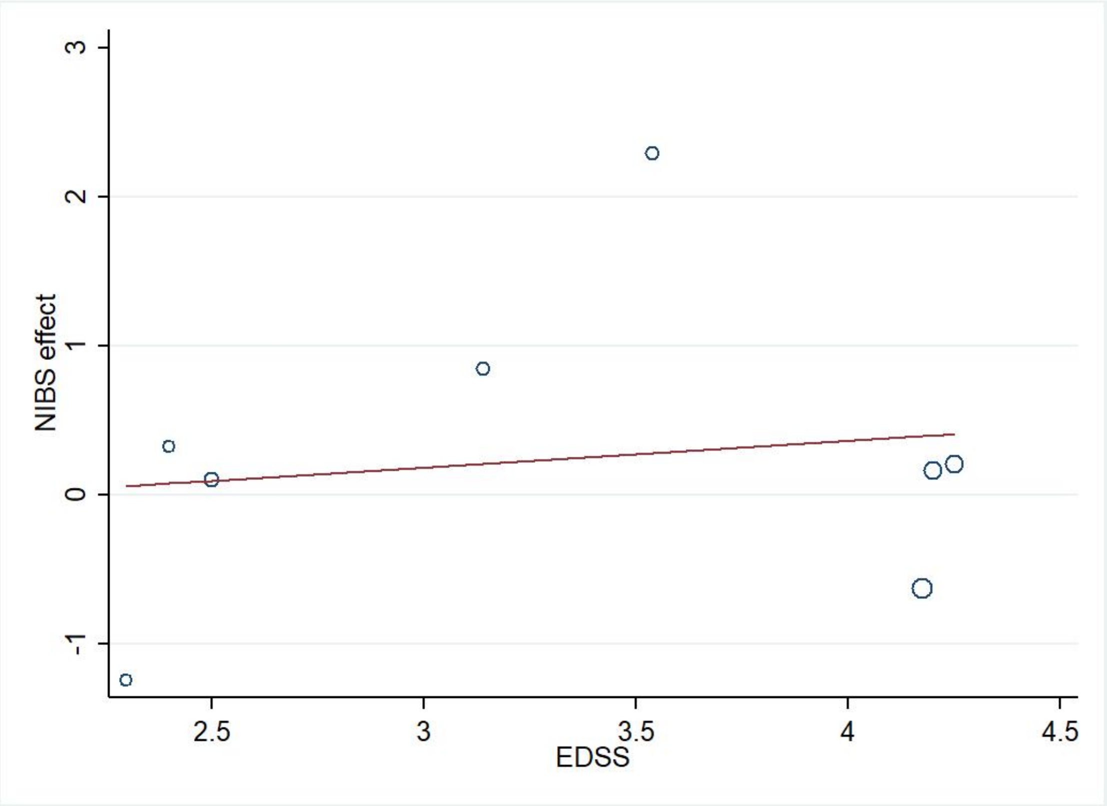

3.3.1.3 Mean Age of Motor

```
. metareg _ES Age , wsse( _seES ) eform graph

Meta-regression                                        Number of obs    =         8
REML estimate of between-study variance                tau2             =         0
% residual variation due to heterogeneity              I-squared_res    =     0.00%
Proportion of between-study variance explained         Adj R-squared    =        .%
With Knapp-Hartung modification
------------------------------------------------------------------------------
         _ES |     exp(b)   Std. Err.      t    P>|t|     [95% Conf. Interval]
-------------+----------------------------------------------------------------
         Age |   .9677419   .0201037    -1.58   0.166     .9197792    1.018206
       _cons |   7.097418    6.59715     2.11   0.080      .730002    69.00439
------------------------------------------------------------------------------
```

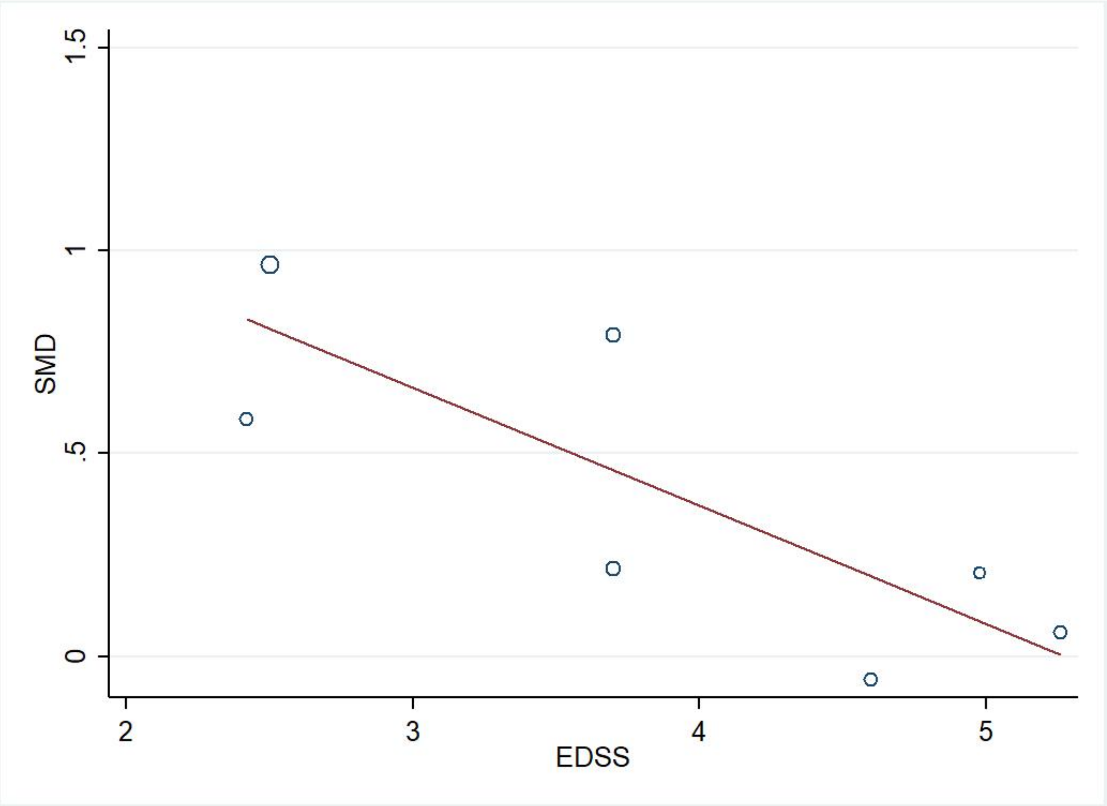

3.3.1.4 Mean EDSS of Motor

```
. metareg _ES EDSS , wsse( _seES ) eform graph

Meta-regression                                        Number of obs   =        7
REML estimate of between-study variance                tau2            =        0
% residual variation due to heterogeneity              I-squared_res   =    0.00%
Proportion of between-study variance explained         Adj R-squared   =       .%
With Knapp-Hartung modification
------------------------------------------------------------------------------
         _ES |     exp(b)   Std. Err.      t    P>|t|     [95% Conf. Interval]
-------------+----------------------------------------------------------------
        EDSS |   .7475328    .1253805    -1.73   0.143     .4857166    1.150476
       _cons |   4.641019    3.031978     2.35   0.066     .8655182    24.88574
------------------------------------------------------------------------------
```

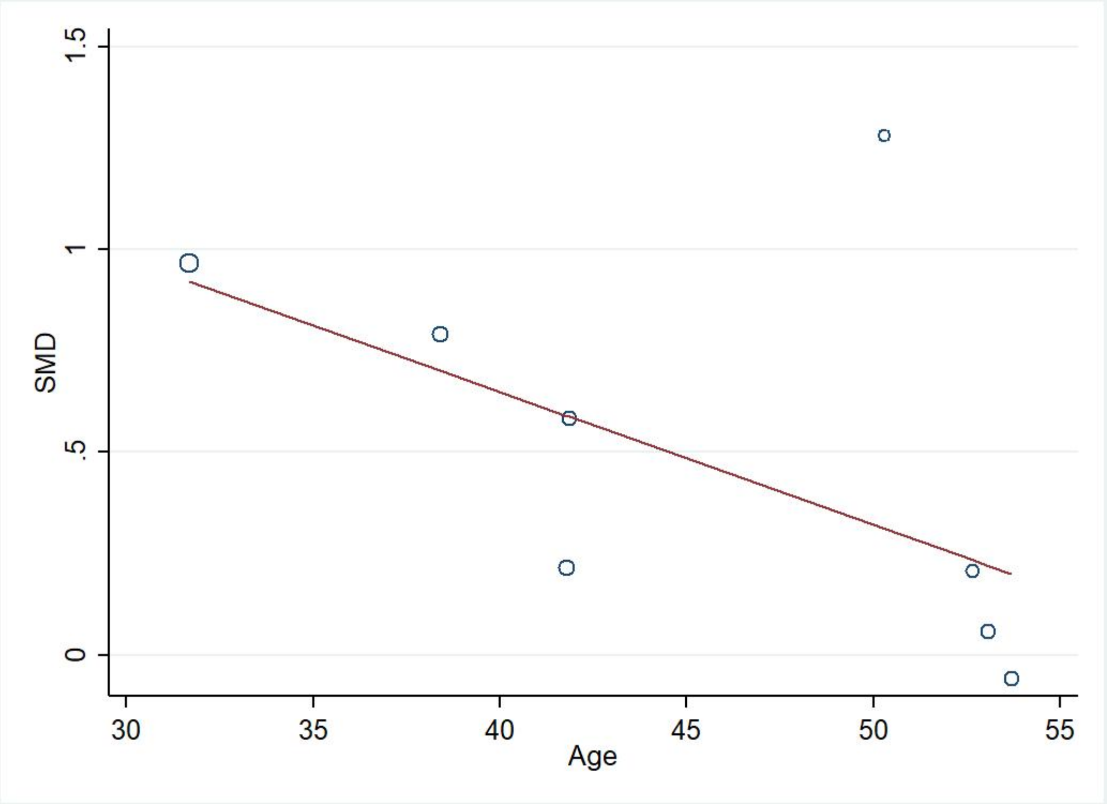

3.4 sensitivity analysis of cognitive-related studies

```
. metan T_E m_E SD_E T_C m_C SD_C, cohen random label(namevar=    Study , yearvar= Year )
```

counts group1(Experimental) group2(Control) texts(220) xlabel(-4,-2,0,2,4)

Study	SMD	[95% Conf.	Interval]	% Weight
Charvet et al. (2018	0.915	0.234	1.596	9.73
Simani et al. (2022)	-0.363	-0.988	0.263	10.01
Hanken et al. (2016)	-0.629	-1.264	0.007	9.96
Fiene et al. (2018)	2.295	1.360	3.230	8.41
Palm et al. (2016)	0.159	-0.535	0.853	9.66
Chalah et al. （DLPFC	-1.241	-2.206	-0.275	8.24
Chalah et al.(PCC) (	-0.465	-1.354	0.425	8.64
Ayache et al. (2016)	0.205	-0.490	0.900	9.66
Grigorescu et al. (2	0.844	-0.075	1.763	8.49
Mattioli et al. (201	0.102	-0.735	0.938	8.92
Salemi et al. (2018)	0.323	-0.636	1.282	8.28
D+L pooled SMD	0.183	-0.320	0.686	100.00

Heterogeneity chi-squared = 45.56 (d.f. = 10) p = 0.000

I-squared (variation in SMD attributable to heterogeneity) = 78.1%

Estimate of between-study variance Tau-squared = 0.5560

Test of SMD=0 : z= 0.71 p = 0.475

3.4.1 Remove Fiene et al.

. metan T_E m_E SD_E T_C m_C SD_C, cohen random label(namevar= Study , yearvar= Year)
counts group1(Experimental) group2(Control) texts(
> 220) xlabel(-4,-2,0,2,4)

Study	SMD	[95% Conf.	Interval]	% Weight
Charvet et al. (2018	0.915	0.234	1.596	11.07
Simani et al. (2022)	-0.363	-0.988	0.263	11.67
Hanken et al. (2016)	-0.629	-1.264	0.007	11.56
Palm et al. (2016)	0.159	-0.535	0.853	10.93
Chalah et al. （DLPFC	-1.241	-2.206	-0.275	8.31
Chalah et al.(PCC) (	-0.465	-1.354	0.425	8.98
Ayache et al. (2016)	0.205	-0.490	0.900	10.92
Grigorescu et al. (2	0.844	-0.075	1.763	8.72

```
Mattioli et al. (201 |    0.102        -0.735       0.938              9.48
Salemi et al. (2018) |    0.323        -0.636       1.282              8.36
---------------------+---------------------------------------------------
D+L pooled SMD       | -0.008        -0.404       0.387             100.00
---------------------+---------------------------------------------------

  Heterogeneity chi-squared =    23.63 (d.f. = 9) p = 0.005
  I-squared (variation in SMD attributable to heterogeneity) =    61.9%
  Estimate of between-study variance Tau-squared =    0.2465

  Test of SMD=0 : z=      0.04 p = 0.966
```

3.4.2 Remove Chalah et al.（DLPFC）

```
. metan T_E m_E SD_E T_C m_C SD_C, cohen random label(namevar=    Study , yearvar= Year )
counts group1(Experimental) group2(Control) texts(
> 220) xlabel(-4,-2,0,2,4)

               Study       |        SMD     [95% Conf. Interval]        % Weight
---------------------+---------------------------------------------------
Charvet et al. (2018 |    0.915          0.234        1.596              12.29
Simani et al. (2022) | -0.363         -0.988        0.263              13.18
Hanken et al. (2016) | -0.629         -1.264        0.007              13.01
Palm et al. (2016)      |    0.159          -0.535        0.853              12.09
Chalah et al.(PCC) ( | -0.465         -1.354        0.425              9.46
Ayache et al. (2016) |    0.205          -0.490        0.900              12.08
Grigorescu et al. (2 |    0.844         -0.075        1.763              9.12
Mattioli et al. (201 |    0.102        -0.735        0.938              10.11
Salemi et al. (2018) |    0.323         -0.636        1.282              8.67
---------------------+---------------------------------------------------
D+L pooled SMD       |    0.098          -0.268        0.465             100.00
---------------------+---------------------------------------------------

  Heterogeneity chi-squared =    16.99 (d.f. = 8) p = 0.030
  I-squared (variation in SMD attributable to heterogeneity) =    52.9%
  Estimate of between-study variance Tau-squared =    0.1633

  Test of SMD=0 : z=      0.53 p = 0.599
```

3.4.3 Remove Charvet et al. (2018）

. metan T_E m_E SD_E T_C m_C SD_C, cohen random label(namevar= Study , yearvar= Year) counts group1(Experimental) group2(Control) texts(
> 220) xlabel(-4,-2,0,2,4)

Study	SMD	[95% Conf. Interval]		% Weight
Simani et al. (2022)	-0.363	-0.988	0.263	16.20
Hanken et al. (2016)	-0.629	-1.264	0.007	15.87
Palm et al. (2016)	0.159	-0.535	0.853	14.21
Chalah et al.(PCC) (	-0.465	-1.354	0.425	10.03
Ayache et al. (2016)	0.205	-0.490	0.900	14.19
Grigorescu et al. (2	0.844	-0.075	1.763	9.55
Mattioli et al. (201	0.102	-0.735	0.938	10.99
Salemi et al. (2018)	0.323	-0.636	1.282	8.94
D+L pooled SMD	-0.033	-0.358	0.292	100.00

Heterogeneity chi-squared = 10.18 (d.f. = 7) p = 0.178
I-squared (variation in SMD attributable to heterogeneity) = 31.3%
Estimate of between-study variance Tau-squared = 0.0679

Test of SMD=0 : z= 0.20 p = 0.843

3.4.4 Remove Hanken et al.

. metan T_E m_E SD_E T_C m_C SD_C, cohen random label(namevar= Study , yearvar= Year) counts group1(Experimental) group2(Control) texts(
> 180) xlabel(-4,-2,0,2,4)

Study	SMD	[95% Conf. Interval]		% Weight
Simani et al. (2022)	-0.363	-0.988	0.263	21.29
Palm et al. (2016)	0.159	-0.535	0.853	17.60
Chalah et al.(PCC) (	-0.465	-1.354	0.425	11.08
Ayache et al. (2016)	0.205	-0.490	0.900	17.57
Grigorescu et al. (2	0.844	-0.075	1.763	10.42

Mattioli et al. (201	0.102	-0.735	0.938	12.45
Salemi et al. (2018)	0.323	-0.636	1.282	9.60
D+L pooled SMD	0.067	-0.237	0.371	100.00

Heterogeneity chi-squared = 6.43 (d.f. = 6) p = 0.377

I-squared (variation in SMD attributable to heterogeneity) = 6.7%

Estimate of between-study variance Tau-squared = 0.0114

Test of SMD=0 : z= 0.43 p = 0.666
